# Prevalence of bias attributable to composite outcome in clinical trials published in 2019–2020: a systematic review

**DOI:** 10.1590/1980-549720250035

**Published:** 2025-06-27

**Authors:** José Mário Nunes da Silva, Juliana Ferreira Souza Conceição, Paula Camila Ramírez, Christian Leonardo Diaz-León, Fredi Alexander Diaz-Quijano

**Affiliations:** I Universidade de São Paulo, School of Public Health, Graduate Program in Epidemiology – São Paulo (SP), Brazil.; II Universidade de São Paulo, School of Public Health – São Paulo (SP), Brazil.; III Universidad Industrial de Santander, School of Physical Therapy – Santander, Bucaramanga, Colombia.; IV Organización Latinoamericana para el Fomento de la Investigación en Salud – Santander, Bucaramanga, Colombia.; V Universidade de São Paulo, School of Public Health, Department of Epidemiology, Laboratório de Inferência Causal em Epidemiologia – São Paulo (SP), Brazil.

**Keywords:** Treatment outcome, Mortality, Bias, Clinical trial, Research methodology, Resultado do tratamento, Mortalidade, Viés, Ensaio clínico, Projetos de pesquisa

## Abstract

**Objective:**

The aim of this study was to investigate the prevalence of bias attributable to composite outcome (BACO) in clinical trials.

**Methods:**

We searched PubMed for randomized clinical trials where the primary outcome was a binary composite that included all-cause mortality among its components from January 1, 2019, to December 31, 2020. For each trial, the BACO index was calculated to assess the correspondence between effects on the composite outcome and mortality. This systematic review was registered in PROSPERO (CRD42021229554).

**Results:**

After screening 1,076 citations and 171 full-text articles, 91 studies were included from 13 different medical areas. The prevalence of significant or suggestive BACO among the 91 included articles was 25.2% (n=23), including 12 with p<0.005 and 11 with p between 0.005 and <0.05. We observed that in 17 (73.9%) of these 23 studies, the BACO index value was between 0 and <1, indicating an underestimation of the effect. The other six studies showed negative values (26.1%), indicating an inversion of the association with mortality. None of the studies showed significant overestimation of the association attributable to the composite outcome.

**Conclusion:**

These findings highlight the need to predefine guidelines for interpreting effects on composite endpoints based on objective criteria such as the BACO index.

## RESUMO

**Objetivo:**

Investigar a prevalência de viés atribuível ao desfecho composto (BACO) em ensaios clínicos.

**Métodos:**

Buscamos no PubMed ensaios clínicos randomizados em que o desfecho primário fosse um composto binário que incluísse mortalidade por todas as causas entre seus componentes, entre 1° de janeiro de 2019 e 31 de dezembro de 2020. Para cada ensaio, o índice BACO foi calculado para avaliar a correspondência entre os efeitos sobre o desfecho composto e aqueles sobre a mortalidade. Esta revisão sistemática foi registrada no PROSPERO (CRD42021229554).

**Resultados:**

Após triagem de 1.076 citações e 171 artigos completos, 91 estudos foram incluídos, abrangendo 13 áreas médicas diferentes. A prevalência de BACO significativo ou sugestivo entre os 91 artigos incluídos foi de 25,2% (n=23), incluindo 12 com p<0,005 e 11 com p entre 0,005 e <0,05. Observamos que em 17 (73,9%) desses 23 estudos, o valor do índice BACO estava entre zero e <1, indicando uma subestimação do efeito. Nos outros seis estudos, os valores foram negativos (26,1%), indicando uma inversão da associação com a mortalidade. Nenhum dos estudos apresentou superestimação significativa da associação atribuível ao desfecho composto.

**Conclusão:**

Esses achados ressaltam a necessidade de pré-definir diretrizes para interpretação dos efeitos em desfechos compostos com base em critérios objetivos, como o índice BACO.

## INTRODUCTION

Composite outcomes are often used in randomized trials to assess the efficacy of a new intervention compared to standard treatment^1,2^. Their use involves analyzing a greater number of outcomes over shorter follow-up periods. Typically, this is expected to increase the power of the study, reduce costs, and provide a quicker response to a research question^3^. However, composite outcomes can lead to misleading conclusions when the individual components, which may vary in importance and frequency, are affected differently or oppositely by the interventions being evaluated^4,5^.

In this context, the Bias Attributable to Composite Outcome (BACO) Index is a recently developed tool that aids in interpreting the effects on a composite outcome^6^. This index corresponds to the ratio between the logarithms of the measures of association for the composite outcome and for mortality. BACO index values different from one indicate that using a composite outcome bias the estimated effect on prognosis as follows: overestimated (BACO index>1), underestimated (BACO index between 0 and <1), or inverted (BACO index<0), using the effect on mortality as the reference point^6^.

Despite the frequent use of composite outcomes, especially in cardiovascular clinical trials, the frequency and direction of BACO have not been widely quantified. Therefore, we aim to investigate the prevalence of BACO in clinical trials published in PubMed between 2019 and 2020.

## METHODS

### Protocol and registration

We conducted the review according to the guidelines of the Preferred Reporting Items for Systematic Reviews and Meta-Analyses (PRISMA)^7^. We registered the protocol in PROSPERO (CRD42021229554).

### Eligibility criteria

#### Inclusion criteria

We included randomized clinical trials where the primary outcome was a binary composite outcome that included all-cause mortality among its components, published in 2019–2020^6^.

#### Exclusion criteria

We excluded cluster-randomized trials, secondary analyses, and subgroup analyses. To ensure precise BACO index estimates, we excluded studies with fewer than five fatal events^6^. Additionally, we excluded four articles that lacked data on the frequency of composite outcomes or mortality.

### Search Strategy

We searched PubMed for articles published electronically in English, Portuguese, or Spanish between January 2019 and December 2020 (updated on April 5, 2021). This decision to limit our search to PubMed was based on its relevance and comprehensive coverage of biomedical literature, particularly in the context of clinical trials using composite outcomes in their analyses, making it an excellent source for identifying these studies. PubMed serves as a practical and widely used resource for clinicians to identify studies of interest in this context. We used the following terms: Composite AND primary AND (endpoint OR outcome OR ("end-point")) AND (mortality OR death) AND (randomized OR randomised) AND (trial). We prioritized a combination of terms that was sufficiently comprehensive and aligned with the approaches used in other relevant literature. Next, the search was exported as a separate file using EndNote Web. This tool enabled the organization and separation of results for each reviewer.

### Selection of studies

Pairs of two independent reviewers screened titles and abstracts of all citations retrieved during the literature search. Subsequently, reviewers read potentially eligible articles in full to determine if they met the eligibility criteria. Any discrepancies between reviewers that arose at each stage of the study selection process were resolved through consensus and, if needed, arbitration by a third reviewer. Prior to both steps of study selection, we conducted a pilot test using random samples of 10 full articles.

### Data extraction and management

We used a predefined standardized protocol where two independent reviewers extracted data from included studies, compared information, and resolved disagreements through discussion. We extracted the following data: article title, year of publication, first author's name, journal published, randomization and blinding process, follow-up period, experimental and control group interventions, sample size in each group, sample loss, components of the composite outcome, number of composite endpoints and deaths, measure of association used, and information on intention-to-treat analysis. We also recorded whether the study's conclusion was based on the composite outcome and if the authors addressed discrepancies between the composite outcome result and mortality, as well as protocol registration on a platform.

### Data analysis

We calculated the BACO index defined as Equation 1 ^6^:


(1) $$BACO\;index=\frac{Ln\left(\varphi_{c}\right)}{Ln\left(\varphi_{d}\right)}$$

Where:

*φ_c_*: relative risk for the composite outcome;

*φ_d_*: relative risk for mortality.

A BACO index equal to one indicates no bias attributable to using a composite outcome, with mortality as the reference measure of association^6^.

We set a predefined significance level of 0.005. However, we used the term suggestive for p-values between 0.005 and 0.05. The 95% confidence intervals for the BACO index and hypothesis testing were conducted following the methods described in the original study^6^. We performed the analyses using Microsoft Office Excel 2019 (Microsoft Corporation, Redmond, Washington).

### Risk-of-bias assessment

For studies suggestive of BACO or with significant BACO, a risk-of-bias assessment was conducted by two independent reviewers. We used the Joanna Briggs Institute (JBI) Critical Appraisal Checklist for RCTs^8^, which includes 13 questions addressing key aspects such as participant selection and allocation, administration of the intervention or exposure, outcome assessment and measurement, participant retention, and the validity of statistical conclusions. Each question in the checklist offers response options: Yes, No, Unclear, or Not Applicable (NA), along with detailed explanations of the assessed criteria. Only "yes" responses contributed to the total score^8^. This additional step aimed to evaluate or rule out the presence of other potential biases in these studies. A third reviewer reassessed discrepancies, and the consensus was reached through discussion. No studies were excluded from the systematic review based on these findings.

## RESULTS

### Search results

**Figure 1:**
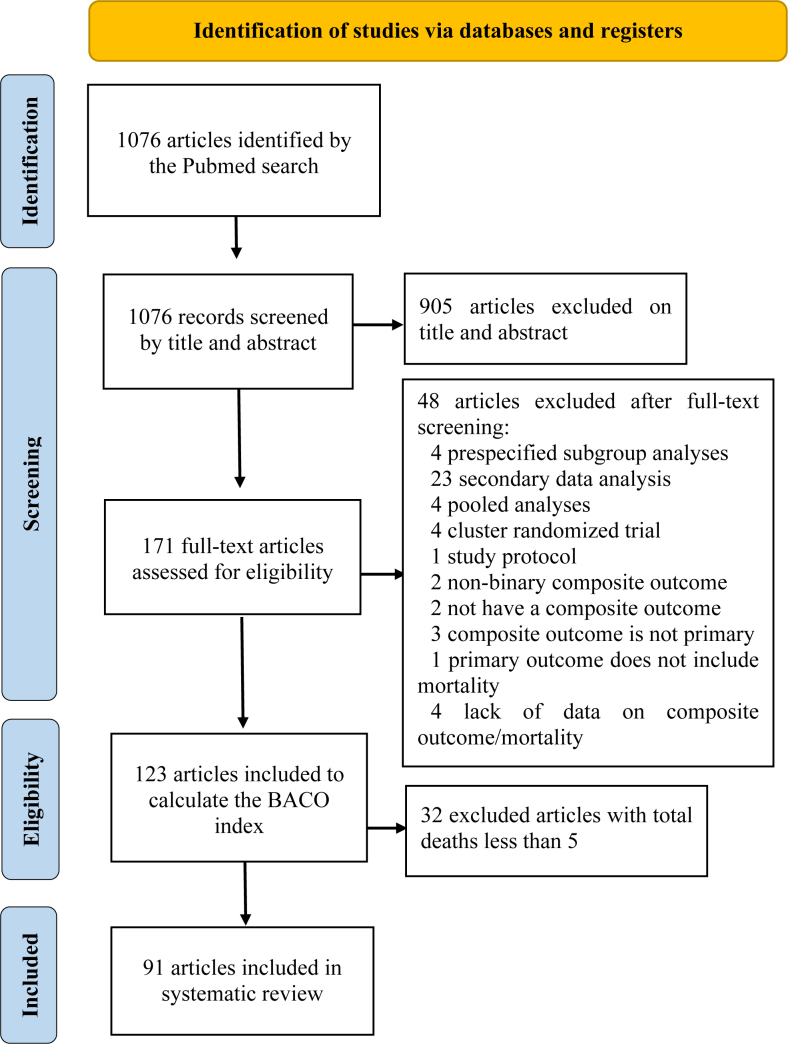
Flowchart of the study identification and selection.

The initial search provided us with a total of 1,076 studies. After reviewing titles and abstracts, we excluded 905 studies. Of the 171 eligible full-text articles, 123 met the study's inclusion criteria. However, we excluded 32 studies due to fewer than 5 total deaths. Ultimately, we selected 91 studies for this review (Figure 1).

### Study characteristics

All articles were written in English and were published in 44 different journals covering 13 different thematic areas, according to Scopus classification. The most prevalent categories were Cardiology and Cardiovascular Medicine (37/91; 40.7%) and Medicine (various, 33/91; 36.2%). The manuscripts were most frequently published in *The New England Journal of Medicine* (17/91; 18.7%), *Circulation* (12/91; 13.2%), and *JAMA* (10/91; 11%) (Table 1).

**Table 1:** Characteristics of the articles included (n=91).

Study characteristic		N	%
Scopus subject category			
	Cardiology and cardiovascular medicine	37	40.7
	Medicine (miscellaneous)	33	36.2
	General medicine	5	5.5
	Pediatrics, perinatology, and child health	4	4.4
	Nephrology	2	2.2
	Critical care and intensive care medicine	2	2.2
	Pulmonary and respiratory medicine	2	2.2
	Others	6	6.6
Publication Journal			
	*The New England Journal of Medicine*	17	18.7
	*Circulation*	12	13.2
	*JAMA*	10	11
	*The Lancet*	5	5.5
	*American Heart Journal*	3	3.3
	*European Heart Journal*	3	3.3
	*Journal of the American College of Cardiology*	3	3.3
	Others	38	41.7
Intention-to-treat analysis			
	Yes	84	92.3
Blinding			
	Yes	76	83.5
Platform protocol registration			
	Yes	84	92.3
Random sequence generation			
	Specific software	64	70.3
	Permuted block randomization	12	13.2
	Random number table	2	2.2
	Raffle	1	1.1
	Unspecified	12	13.2

The majority (84/91; 92.3%) of included articles had registered protocols, and the same number conducted intention-to-treat analysis. Masked intervention was evaluated in most studies (76/91; 83.5%), and allocation was based on specific software randomization sequences in 64/91 studies (70.3%). Six articles reported discrepancies between the composite outcome association and mortality (6/91; 6.6%), and conclusions were not based on the composite outcome in two manuscripts (2/91; 2.2%).

### Prevalence of BACO

Out of the 91 included articles, 23 (25.2%) had a significant or suggestive BACO, including 12 with p<0.005 and 11 with p between 0.005 and <0.05 (Tables 2 and 3). These 23 studies included a total of 21,285 participants in the experimental group, with sample sizes ranging from 46 to 5,523 patients, a median of 417 (IQR=182–1,050); presenting 24 to 975 composite outcomes, with a median of 89 (IQR=69–203); and 2–119 deaths, with a median of 15 (IQR=7–36). In the control groups, there were a total of 20,048 participants, with sample sizes ranging from 54 to 5,493, a median of 409 (IQR=190–1,038). In these groups, the number of composite events ranged from 19 to 924, with a median of 88 (IQR=60–176); and fatal events ranged from 1 to 100, with a median of 11 (IQR=6–38) (Table 2).

**Table 2:** General description of the study population and outcomes of clinical trials selected (n=23).

Authors	Composite outcome description besides death	Composite/deaths	
		Intervention group	Control group
Dangas et al.^9^	Thromboembolic events.	105/64	78/38
Yasuda et al.^10^	Stroke, systemic embolism, myocardial infarction, unstable angina requiring revascularization.	89/41	121/73
Schuetz et al.^11^	Admission to the intensive care unit from the medical ward; non-elective hospital re-admission after discharge; major complications from admission to day 30.	232/73	272/100
Stone et al.^12^	Stroke, or MI at 3 years.	203/119	176/89
Vardeny et al.^13^	Hospitalization for cardiovascular or pulmonary causes during each enrolling influenza season, with censoring in the first 2 weeks after vaccination and after July 31 of the respective season.	975/92	924/78
Lanz et al.^14^	Any stroke, life-threatening or disabling bleeding, major vascular complications, coronary artery obstruction requiring intervention, acute kidney injury, rehospitalization for valve-related symptoms, or congestive heart failure	87/9	60/3
Lomivorotov et al.^15^	Nonfatal MI, need for extracorporeal membrane oxygenation, cardiopulmonary resuscitation, acute kidney injury, prolonged mechanical ventilation, and neurologic event.	74/2	91/4
Vermeersch et al.^16^	Treatment with systemic corticosteroids and/or antibiotics for respiratory reasons, step-up in hospital for respiratory reasons.	69/3	86/6
Frith et al.^17^	Survival time to hospitalization, residential care admission.	111/29	84/11
Wilson et al.^18^	Lung transplant, or first non-elective hospital admission for any reason.	84/24	80/18
Koch et al.^19^	Multisystem organ failure, cardiac, pulmonary, neurologic, renal failure, infection, gastrointestinal, any reoperation, and vascular events.	553/15	594/23
Willems et al.^20^	Unplanned hospitalization for either symptomatic ventricular tachyarrhythmia or worsening heart failure.	25/6	23/2
Futier et al.^21^	Acute kidney injury, acute respiratory failure requiring mechanical ventilation, acute heart failure, major septic complications, and unplanned reoperation 14 days after surgery.	139/36	362/27
Onland et al.^22^	Bronchopulmonary dysplasia at 36 weeks’ postmenstrual age.	128/28	140/45
Thiele et al.^23^	Stroke, MI, infection requiring antibiotic treatment, and acute kidney injury.	59/7	58/5
Karaye et al.^24^	Persistence of HF symptoms, unrecovered LVEF (less than 55%).	36/3	43/9
Lee et al.^25^	MI, stroke, or any revascularization.	93/15	89/21
Araújo et al.^26^	Preterm birth <37 weeks’ gestation or NICU admission <28 days after birth, or small for gestational age birthweight <3rd percentile.	75/4	76/10
Zhang et al.^27^	Myocardial infarction.	39/19	19/8
De Luca et al.^28^	MI, stent thrombosis, stroke, target vessel revascularization, and bleeding	85/23	88/16
Tong et al.^29^	ACS, ischemia-driven urgent revascularization, and non-cardioembolic ischemic stroke.	24/8	38/1
Sekiziyivu et al.^30^	Virological failure, treatment-limiting ART toxic effects, and LTFU.	284/11	314/8
Johnston et al.^31^	Stroke.	303/36	362/27

MI: myocardial infarction; HF: heart failure; LVEF: left ventricular ejection fraction; NICU: neonatal intensive care unit; ACS: acute coronary syndrome; ART: antiretroviral therapy; LTFU: Loss to follow-up.

**Table 3:** Relative risks of composite and death and BACO index in clinical trials (n=23).

Authors	RR_c_ (95%CI)	RR_d_ (95%CI)	BACO index (95%CI)	p-value^a^
Dangas et al.^9^	1.33 (1.01–1.76)	1.67 (1.13–2.46)	0.56 (0.18–0.95)	0.025
Yasuda et al.^10^	0.74 (0.57–0.95)	0.56 (0.39–0.82)	0.53 (0.21–0.86)	0.004
Schuetz et al.^11^	0.84 (0.72–0.98)	0.72 (0.54–0.96)	0.52 (0.07–0.98)	0.038
Stone et al.^12^	1.16 (0.97–1.40)	1.35 (1.04–1.75)	0.51 (0.07–0.94)	0.025
Vardeny et al.^13^	1.06 (0.98–1.13)	1.18 (0.88–1.59)	0.33 (-0.31–0.96)	0.038
Lanz et al.^14^	1.43 (1.06–1.92)	2.96 (0.81–10.85)	0.33 (-0.10–0.76)	0.002
Lomivorotov et al.^15^	0.84 (0.66–1.06)	0.52 (0.10–2.78)	0.27 (-0.45–0.99)	0.045
Vermeersch et al.^16^	0.84 (0.67–1.05)	0.52 (0.13–2.06)	0.27 (-0.35–0.88)	0.019
Frith et al.^17^	1.28 (1.22–1.35)	2.56 (1.35–4.83)	0.26 (0.08–0.45)	<0.0001
Wilson et al.^18^	1.07 (0.86–1.33)	1.36 (0.77–2.40)	0.22 (-0.46–0.90)	0.024
Koch et al.^19^	0.93 (0.84–1.02)	0.65 (0.34–1.24)	0.17 (-0.14–0.49)	<0.0001
Willems et al.^20^	1.21 (0.76–1.95)	3.35 (0.70–16.11)	0.16 (-0.22–0.55)	<0.0001
Futier et al.^21^	1.11 (0.91–1.36)	2.00 (0.76–5.29)	0.16 (-0.17–0.48)	<0.0001
Onland et al.^22^	0.95 (0.84–1.08)	0.65 (0.42–0.99)	0.11 (-0.17–0.39)	<0.0001
Thiele et al.^23^	1.03 (0.76–1.41)	1.42 (0.46–4.40)	0.09 (-0.77–0.94)	0.036
Karaye et al.^24^	0.98 (0.80–1.20)	0.39 (0.11–1.36)	0.02 (-0.20–0.23)	<0.0001
Lee et al.^25^	1.00 (0.77–1.29)	0.68 (0.36–1.30)	0.01 (-0.66–0.67)	0.003
Araújo et al.^26^	1.02 (0.76–1.36)	0.41 (0.13–1.30)	-0.02 (-0.35–0.32)	<0.0001
Zhang et al.^27^	1.03 (0.60–1.78)	0.57 (0.22–1.46)	-0.06 (-1.07–0.96)	0.040
De Luca et al.^28^	0.95 (0.72–1.26)	1.42 (0.76–2.66)	-0.14 (-1.08–0.80)	0.017
Tong et al.^29^	0.64 (0.39–1.04)	8.06 (1.01–64.15)	-0.22 (-0.57–0.14)	<0.0001
Sekiziyivu et al.^30^	0.91 (0.80–1.04)	1.39 (0.56–3.44)	-0.27 (-1.16–0.62)	0.005
Johnston et al.^31^	0.83 (0.72–0.97)	1.33 (0.81–2.18)	-0.65 (-2.04–0.74)	0.020

RR_c_: relative risk for the composite outcome; RR_d_: relative risk for any-cause death; CI, confidence interval; BACO, bias attributable to composite outcomes; BACO index: *Ln (RRc)/Ln (RR*_d_ *)*;

a *P-value* for the null hypothesis of the BACO index is equal to 1.

We observed that in 17 (73.9%) of these 23 studies, the BACO index value was between 0 and <1; the remaining six had negative values, indicating an inversion of the association concerning a fatal event (mortality). None of the studies showed significant overestimation of the association attributable to the composite outcome (Table 3). More information on all 91 included articles can be found in the Supplementary Material (https://doi.org/10.17605/OSF.IO/TY73W).

### Methodological quality of included studies

Regarding the JBI checklist, the 23 studies with significant or suggestive BACO scored between 9 and 13 (Table 4). In total, 14 studies achieved the maximum score by meeting all 13 criteria of quality^11–16,18,19,21,22,25,26,29,31^. In nine studies, participants or the personnel delivering the treatment were not blinded to group assignment^9,10,17,20,23,24,27,28,30^. In three studies, it was unclear whether the outcome assessors were blinded to treatment allocation^17,27,30^. Additionally, in two studies, it was not possible to determine whether allocation to groups was adequately concealed^17,24^. Finally, one study showed baseline differences between the groups^17^.

**Table 4:** Assessment of the quality of the 23 studies with significant or suggestive BACO.

Authors	Critical Appraisal Questions													Total score
	D1			D2			D3			D4	D5			
	1	2	3	4	5	6	7	8	9	10	11	12	13	
Dangas et al.^9^	Y	Y	Y	N	N	Y	Y	Y	Y	Y	Y	Y	Y	11
Yasuda et al.^10^	Y	Y	Y	N	N	Y	Y	Y	Y	Y	Y	Y	Y	11
Schuetz et al.^11^	Y	Y	Y	Y	Y	Y	Y	Y	Y	Y	Y	Y	Y	13
Stone et al.^12^	Y	Y	Y	Y	Y	Y	Y	Y	Y	Y	Y	Y	Y	13
Vardeny et al.^13^	Y	Y	Y	Y	Y	Y	Y	Y	Y	Y	Y	Y	Y	13
Lanz et al.^14^	Y	Y	Y	Y	Y	Y	Y	Y	Y	Y	Y	Y	Y	13
Lomivorotov et al.^15^	Y	Y	Y	Y	Y	Y	Y	Y	Y	Y	Y	Y	Y	13
Vermeersch et al.^16^	Y	Y	Y	Y	Y	Y	Y	Y	Y	Y	Y	Y	Y	13
Frith et al.^17^	Y	U	N	N	N	U	Y	Y	Y	Y	Y	Y	Y	9
Wilson et al.^18^	Y	Y	Y	Y	Y	Y	Y	Y	Y	Y	Y	Y	Y	13
Koch et al.^19^	Y	Y	Y	Y	Y	Y	Y	Y	Y	Y	Y	Y	Y	13
Willems et al.^20^	Y	Y	Y	N	N	Y	Y	Y	Y	Y	Y	Y	Y	11
Futier et al.^21^	Y	Y	Y	Y	Y	Y	Y	Y	Y	Y	Y	Y	Y	13
Onland et al.^22^	Y	Y	Y	Y	Y	Y	Y	Y	Y	Y	Y	Y	Y	13
Thiele et al.^23^	Y	Y	Y	N	N	Y	Y	Y	Y	Y	Y	Y	Y	11
Karaye et al.^24^	Y	U	Y	N	N	Y	Y	Y	Y	Y	Y	Y	Y	10
Lee et al.^25^	Y	Y	Y	Y	Y	Y	Y	Y	Y	Y	Y	Y	Y	13
Araújo et al.^26^	Y	Y	Y	Y	Y	Y	Y	Y	Y	Y	Y	Y	Y	13
Zhang et al.^27^	Y	Y	Y	N	N	U	Y	Y	Y	Y	Y	Y	Y	10
De Luca et al.^28^	Y	Y	Y	N	N	Y	Y	Y	Y	Y	Y	Y	Y	11
Tong et al.^29^	Y	Y	Y	Y	Y	Y	Y	Y	Y	Y	Y	Y	Y	13
Sekiziyivu et al.^30^	Y	Y	Y	N	N	U	Y	Y	Y	Y	Y	Y	Y	10
Johnston et al.^31^	Y	Y	Y	Y	Y	Y	Y	Y	Y	Y	Y	Y	Y	13

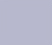
 Y = Yes; 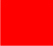 N = No; 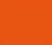 U = Unclear.

*Note:* Domain 1 (D1) - Selection and allocation; Domain 2 (D2) - Administration of intervention/exposure; Domain 3 (D3) - Assessment, detection, and measurement of the outcome; Domain 4 (D4) - Participant retention; and Domain 5 (D5) - Statistical conclusion validity. The question in each column is: 1. Was true randomization used for assignment of participants to treatment groups? 2. Was allocation to groups concealed? 3. Were treatment groups similar at the baseline? 4. Were participants blind to treatment assignment? 5. Were those delivering the treatment blind to treatment assignment? 6. Were treatment groups treated identically other than the intervention of interest? 7. Were outcome assessors blind to treatment assignment? 8. Were outcomes measured in the same way for treatment groups? 9. Were outcomes measured in a reliable way? 10. Was follow-up complete and, if not, were differences between groups in terms of their follow-up adequately described and analyzed? 11. Were participants analyzed in the groups to which they were randomized? 12. Was appropriate statistical analysis used? 13. Was the trial design appropriate and any deviations from the standard RCT design (individual randomization, parallel groups) accounted for in the conduct and analysis of the trial?

## DISCUSSION

In our study, a quarter of the articles selected for review had a significant or suggestive BACO index. In all these cases, the BACO was significantly <1, indicating that the use of composite outcomes underestimated the association between the intervention and the prognosis, and in some cases even inverted the association.

The underestimation of the effect can be interpreted as a dilution of the association due to the inclusion of events affected differently by the intervention^6^. This phenomenon leads to a contradictory situation because composite outcomes are often used to obtain a higher number of events and, thus, greater statistical power. However, if the use of composite outcomes leads to a dilution of the effect, it paradoxically results in a reduction of the study's power.

This explains situations such as the one observed in the study by Onland et al.^22^, where hydrocortisone therapy was associated with a significant reduction in mortality among premature infants, while the effect on the composite outcome was not statistically significant (Table 3). Another recently recognized example, though beyond the scope of this review, involved the use of a composite outcome that underestimated the effect of cocoa extract supplementation on prognosis. While the intervention did not reduce the incidence of the first cardiovascular event, it was associated with a 27% reduction in mortality from that cause^32^.

In another direction, underestimation can also lead to the failure to identify a harmful effect of the intervention. This occurred in the study by Stone et al.^12^, where percutaneous coronary intervention (PCI) significantly increased mortality compared to coronary artery bypass grafting, while the association with the composite outcome was not statistically significant.

In other cases, the underestimation of the effect does not qualitatively change the conclusions but can lead to quantitatively significant differences, as seen in the study by Dangas et al.^9^. In this study, the effect of rivaroxaban was evaluated, finding that the excess risk for mortality was 67%. In contrast, the excess risk for the composite outcome was only 33%.

In six of the studies with a significant or suggestive BACO, the index value was negative, indicating an inversion of the association between the composite outcome and mortality. Beyond our review, we found an example of this trend in the study by Laurens et al.^33^, which evaluated the effect of stopping prophylactic use of cotrimoxazole in adults with human immunodeficiency virus (HIV) infection. The authors compared standard prophylaxis using cotrimoxazole with its discontinuation, either alone or combined with chloroquine. The associations of the primary and secondary composite outcomes were in the opposite direction to mortality, with BACO indices of −0.48 (95%CI −1.79–0.83; p=0.03) and −0.69 (95%CI −2.31–0.93; p=0.04) for the primary and secondary composite outcomes, respectively^34^. This finding suggests that caution should be exercised when interpreting results, as they include effects in opposite directions. In such cases, it would be more appropriate to evaluate and interpret the components of the composite outcome individually.

There are some recommendations for constructing a composite outcome, such as considering the relevance of the outcomes for the patient, the frequency of the components of the composite outcome, and the similarity of the treatment effects across events^3^. However, designing a composite outcome that both accurately reflects prognosis and enables efficient evaluation of intervention effects remains challenging. In this context, the BACO index provides the possibility to test and guide interpretation, which can be predefined without affecting the study's objectivity.

Among the studies identified as suggestive or significant for BACO, we observed that these were generally high-quality clinical trials based on the JBI checklist^8^. This suggests that biases in conclusions related to composite outcomes may occur independently of overall methodological quality and may not be detected by conventional risk-of-bias tools. These findings support the use of the BACO index as a complementary approach for assessing the validity of composite outcomes as prognostic indicators.

We therefore recommend using the BACO index to guide decisions about the adoption of composite outcomes. If the index does not significantly differ from the null value, it suggests no statistical evidence against using the composite outcome as a prognostic measure. Conversely, a BACO index significantly different from 1 indicates a potential distortion in the effect estimate, warranting disaggregation of the outcome components to allow for a more detailed and accurate interpretation^6,34^.

As limitations, our study is restricted to a relatively short time period and included primarily studies initiated before the pandemic. This shorter time frame allowed us to conduct a more detailed and focused analysis, ensuring additional methodological rigor in the evaluation of the included studies. Nevertheless, we consider it relevant to continue the study by expanding the time period and databases to account for the variability across other areas of knowledge and assess the determinants of bias. Therefore, we cannot infer that the prevalence and direction of BACO indices will be similar in other scenarios. However, a recent study focusing on clinical trials in COVID-19 patients estimated the BACO index in 28 effect estimates on composite outcomes^35^. In most studies, the composite outcome estimate was closer to the null value than that of mortality, and the BACO index was significantly <1 in five studies. Similar to the present study, there was no statistically significant overestimation of the effect associated with composite outcomes. Thus, we consider that in various scenarios, underestimation of the effect is likely the most prevalent bias resulting from the use of composite outcomes.

In conclusion, we observed suggestive results or statistically significant BACO in a quarter of the selected studies. In most cases, the bias consisted of underestimating the effects, and in others, there was an inversion of the direction of the RR. These findings highlight the need to predefine guidelines for interpreting effects on composite endpoints based on objective criteria, such as the BACO index.

## Supplementary Material





## Data Availability

The database used to calculate the BACO index is available in the supplementary material. Additional information is available from the corresponding author on request.
