# Supplementary material for: Prevalence of bias attributable to composite outcome in clinical trials published in 2019–2020: a systematic review
Source: Rev Bras Epidemiol. 2025 Jun 27;28:e250035. doi: 10.1590/1980-549720250035 (PMC12204237; doi:10.1590/1980-549720250035)
Supplement: Supplementary file 1 [file 1980-5497-rbepid-28-e250035-suppl1.pdf]

Unique ID	D1	D2	D3	D4	D5	Overall
Koch et al.	+	+	+	+	+	+
Lanz et al.	+	+	+	+	+	+
Lomivorotov et al.	+	+	+	+	+	+
Onland et al.	!	+	+	+	+	!
Sekiziyivu et al.	+	!	+	!	+	!
Schuetz et al.	+	+	+	+	+	+
Stone et al.	+	+	+	+	+	+
Futier et al.	+	+	+	+	+	+
Yasuda et. al	!	+	+	+	+	!
Araújo et al.	+	+	+	+	!	!
Dangas et al.	!	+	+	+	+	!
De Luca et al.	!	+	+	+	+	!
Frith et al.	-	+	+	-	+	-
Johnston et al.	+	+	+	+	+	+
Karaye et al.	!	+	+	+	+	!
Lee et al.	+	+	+	+	+	+
Thiele et al.	!	+	+	+	+	!
Tong et al	+	+	+	+	!	!
Vardeny et al	+	+	+	+	+	+
Vermeersch et al	+	+	+	+	+	+
Willems et al.	+	!	+	+	+	!
Wilson et al.	+	+	+	+	+	+
Zhang et al.	!	+	+	+	+	!

+ Low risk
! Some concerns
- High risk

D1 Randomisation process
D2 Deviations from the intended interventions
D3 Missing outcome data
D4 Measurement of the outcome
D5 Selection of the reported result

Figure S1. Assessment of bias risk in the 23 studies with significant or suggestive BACO.

Supplementary Table S1 - Information on the 91 articles included in the study

Study ID	Citation	Title	Journal	doi	Intervention	Control	Definition of composite outcome	Follow-up time	Experimental group (N)	Control group (N)	Composite outcomes - experimental group (N)	Composite outcomes - control group (N)	Death - experimental group (N)	Death - control group (N)	RRc	RRd	BACO	95%CI lower	95%CI higher	p-value	Measure of association in the study	Intent-to-treat analysis	Study protocol registry	Protocol link
1	Iversen *et al.* 2019	Partial Oral versus Intravenous Antibiotic Treatment of Endocarditis	The New England Journal of Medicine	10.1056/NEJMoa1808312	Intravenous antibiotics to continue intravenous treatment	Intravenous antibiotics to switch to oral antibiotic treatment	**Page 3:** 'Composite of all-cause mortality, unplanned cardiac surgery, clinically evident embolic events, or relapse of bacteremia with the primary pathogen (detected in blood cultures obtained during follow-up or for clinical reasons) from randomization through 6 months after antibiotic treatment was completed.'	6 months	201	199	18	24	7	13	0.74	0.53	0.47	-0.23	1.18	0.1448	Risk difference and Hazard Ratio	Yes	Yes	https://clinicaltrials.gov/ct2/show/NCT01375257
2	Katheria *et al.* 2019	Association of Umbilical Cord Milking vs Delayed Umbilical Cord Clamping With Death or Severe Intraventricular Hemorrhage Among Preterm Infants	JAMA	10.1001/jama.2019.16004	Umbilical cord milking was performed by having the obstetrician hold the infant below the level of the cesarean incision (or below the level of the introitus for vaginal delivery) and 20 cm of the umbilical cord	Delayed Umbilical Cord Clamping	**Page 1880:** 'The primary outcome of the trial was incidence of death or severe intraventricular hemorrhage at 6 months' corrected gestational age. Severe intraventricular hemorrhage was categorized as grade 3 or grade 4.'	6 months	236	238	29	20	17	15	1.46	1.14	2.84	-8.43	14.12	0.7485	Risk difference	No	Yes	https://clinicaltrials.gov/ct2/show/NCT03019367
3	Holm *et al.* 2020	Percutaneous coronary angioplasty versus coronary artery bypass grafting in the treatment of unprotected left main stenosis: updated 5-year outcomes from the randomised, non-inferiority NOBLE trial	The Lancet	10.1016/S0140-6736(19)32972-1	Percutaneous coronary angioplasty in the treatment of unprotected left main stenosis	Coronary artery bypass grafting in the treatment of unprotected left main stenosis	**Page 3:** 'Composite of MACCE, comprising all-cause mortality, non-procedural myocardial infarction, repeat revascularisation, or stroke, assessed after 5 years.'	5 years	598	603	165	110	54	50	1.51	1.09	4.85	-14.72	24.43	0.6997	Hazard Ratio	Yes	Yes	https://clinicaltrials.gov/ct2/show/NCT01496651
4	Locatelli *et al.* 2019	Cardiovascular Safety and All-Cause Mortality of Methoxy Polyethylene Glycol-Epoetin Beta and Other Erythropoiesis-Stimulating Agents in Anemia of CKD: A Randomized Noninferiority Trial	Clinical Journal of the American Society of Nephrology	10.2215/CJN.01380219	Methoxy polyethylene glycol-epoetin beta dose was 0.6 mg/kg and maintenane administered at monthly intervals with a starting dose of 120, 200, or 360 mg	Reference agent (epoetin alfa/beta or darbepoetin).	**Page 2:** 'Composite end point was the time to the first occurrence of death, nonfatalmyocardial infarction, or nonfatal stroke'	8.5 years	1409	1409	640	644	558	557	0.99	1.00	-3.47	#	214.07	0.9678	Hazard Ratio	Yes	Yes	https://clinicaltrials.gov/ct2/show/NCT00773513
5	Packer *et al.* 2019	Effect of Catheter Ablation vs Antiarrhythmic Drug Therapyon Mortality, Stroke, Bleeding, and Cardiac Arrest Among Patients With Atrial Fibrillation: The CABANA Randomized Clinical Trial	JAMA	10.1001/jama.2019.0693	Catheter ablation underwent pulmonary vein isolation and were treated with anticoagulation for at least 3 months after the ablation	Antiarrhythmic Drug Therapy	**Page 11**: 'Composite end point of death, disabling stroke, serious bleeding, or cardiac arrest.'	48.5 months	1108	1096	89	101	58	67	0.87	0.86	0.89	-0.31	2.08	0.8506	Hazard Ratio	Yes	Yes	https://clinicaltrials.gov/ct2/show/NCT00911508
6	Kozhuharov *et al* . 2019	Effect of a Strategy of Comprehensive Vasodilation vs Usual Care on Mortality and Heart Failure Rehospitalization Among Patients With Acute Heart Failure: The GALACTIC Randomized Clinical Trial	JAMA	10.1001/jama.2019.18598	Early intensive and sustained vasodilation was a comprehensive pragmatic approach of maximal and sustained vasodilation combining individualized doses of sublingual and transdermal nitrates, low-dose oral hydralazine for 48 hours, and rapid up-titration of angiotensin-converting enzyme inhibitors, [illegible]	Usual Care	**Page 2294**: 'Composite of all-cause mortality or rehospitalization for AHFat 180 days'	180 days	386	402	117	111	55	61	1.10	0.94	-1.48	-12.01	9.05	0.6440	Hazard Ratio	Yes	Yes	https://clinicaltrials.gov/ct2/show/NCT00512759
7	Blackwell *et al.* 2020	17-OHPC to Prevent Recurrent Preterm Birth in Singleton Gestations (PROLONG Study): A Multicenter, International, Randomized Double-Blind Trial	American Journal of Perinatology	10.1055/s-0039-3400227	The 17-OHPC (hydroxyprogesterone caproate, 250mg/ mL), benzyl benzoate, castor oil, and benzyl alcohol to Prevent Recurrent Preterm Birth in Singleton Gestations	Placebo	PTB<35 weeks (all deliveries occurring from randomization until 350/7 weeks of gestation, including miscarriages or abortions occurring from 160/7 through 196/7 weeks) and a composite neonatal morbidity and mortality index. The composite index included any of the following: neonatal death, grade-3 or-4 intraventricular hemorrhage, respiratory distress syndrome, bronchopulmonary dysplasia, necrotizing enterocolitis, or proven sepsis.	36 weeks	1130	578	61	28	6	3	1.11	1.02	4.76	-278.99	288.51	0.9793	Relative Risk	Yes	Yes	https://clinicaltrials.gov/ct2/show/NCT01004029
8	Schuetz *et al* . 2019	Individualised nutritional support in medical inpatients at nutritional risk: a randomised clinical trial	The Lancet	10.1016/S0140-6736(18)32776-4	Protocol-guided individualised nutritional support to reach protein and caloric goals.	Standard hospital food	**Page 4:** 'The composite primary endpoint was defined as adverse clinical outcome within 30 days, and it included: all-cause mortality; admission to the intensive care unit from the medical ward; non-elective hospital re-admission after discharge; major complications as a new occurrence including adjudicated nosocomial infection, respiratory failure, a major cardiovascular event (ie, stroke, intracranial bleeding, cardiac arrest, myocardial infarction, or pulmonary [illegible]	30 days	1050	1038	232	272	73	100	0.84	0.72	0.52	0.07	0.98	0.0389	Odds Ratio	Yes	Yes	https://clinicaltrials.gov/ct2/show/NCT02517476
9	Graham *et al.* 2019	Corticosteroid Therapy in Neonates Undergoing Cardiopulmonary Bypass	Journal of the American College of Cardiology	10.1016/j.jacc.2019.05.060	Intraoperative methylprednisolone in neonates undergoing cardiac surgery with cardiopulmonary bypass.	Placebo	**Page 661:** Composite of death and objective signs of inadequate cardiac output that has been validated in neonates undergoing cardiac surgery with CPB'	36 hours	91	99	27	40	5	6	0.73	0.91	3.15	-32.66	38.96	0.9064	Odds Ratio	Yes	Yes	https://clinicaltrials.gov/ct2/show/NCT03229538
10	Basu *et al* . 2019	Oral vitamin A supplementation in very low birth weight neonates: a randomized controlled trial	European Journal of Pediatrics	10.1007/s00431-019-03412-w	Vitamin A bottle contained 10,000 IU of retinol/mL supplementation in very low birth weight neonates.	Placebo	**Page 1257:** 'Composite incidence of all-cause mortality and/or oxygen requirement for 28 days, measured at day 28 of life.'	28 days	98	98	11	25	9	16	0.44	0.56	1.43	0.31	2.54	0.4529	Relative Risk	Yes	Yes	http://ctri.nic.in/Clinicaltrials/advsearch.php
11	Lomivorotov *et al.* 2020	Effect of Intraoperative Dexamethasone on Major Complications and Mortality Among Infants Undergoing Cardiac Surgery The DECISION Randomized Clinical Trial	JAMA	10.1001/jama.2020.8133	Dexamethasone group received 1mg/kg of dexamethasone Among Infants Undergoing Cardiac Surgery	0.9% sodium chloride	**Page 2487**: 'Composite of death, nonfatal myocardial infarction, need for extracorporeal membrane oxygenation, cardiopulmonary resuscitation, acute kidney injury (at least stage "Injury" according to the pediatric Risk, Injury, Failure, Loss, End Stage Renal Disease [pRIFLE] scale),16 prolonged mechanical ventilation (≥24 hours), and neurologic event (stroke, seizures, or coma).'	30 days	194	200	74	91	2	4	0.84	0.52	0.27	-0.45	0.99	0.0454	Hazard Ratio	Yes	Yes	https://clinicaltrials.gov/ct2/show/NCT02615262
12	Vermeersch *et al* . 2019	Azithromycin during Acute COPD Exacerbations Requiring Hospitalization (BACE): a Multicentre, Randomized, Double-blind, Placebo-controlled Trial	American Journal of Respiratory and Critical Care Medicine	10.1164/rccm.201901-0094OC	Azithromycin in the acute treatment of chronic obstructive pulmonary disease	Placebo	**Page 5:** 'The primary endpoint was the TF rate within 90 days analyzed using time-to-first event methods, with TF defined as the composite of 3 endpoints: (1) treatment intensification (TI) with systemic corticosteroids and/or antibiotics for respiratory reasons, (2) step-up in hospital care (SH) including transfer to the intensive care unit (ICU) or readmission for respiratory reasons or (3) all-cause mortality.'	3 months	147	154	69	86	3	6	0.84	0.52	0.27	-0.35	0.88	0.0196	Hazard Ratio	Yes	Yes	https://clinicaltrials.gov/ct2/show/NCT02135354
13	Frith *et al.* 2020	Pragmatic randomised controlled trial of a personalized intervention for carers of people requiring home oxygen therapy	Chronic Respiratory Disease	10.1177/1479973119897277	Personalised educational sessions focusing on motivating carers to take actions to assist patients that requiring home oxygen therapy	Usual Care	**Page 3:** 'Composite of patients survival time to hospitalisation, residential care admission or death over the course of 12 months.'	12 months	100	97	111	84	29	11	1.28	2.56	0.26	0.08	0.45	0.0000	Hazard Ratio	Yes	Yes	https://anzctr.org.au/Trial/Registration/TrialReview.aspx?id=81873&isReview=true
14	Lanz *et al* . 2019	Safety and efficacy of a self-expanding versus a balloon-expandable bioprosthesis for transcatheter aortic valve replacement in patients with symptomatic severe aortic stenosis: a randomised non-inferiority trial	The Lancet	10.1016/S0140-6736(19)32220-2	the ACURATE neo bioprosthesis, which combines a self-expanding nitinol frame with three porcine pericardial leaflets, and a stent body with an outer and inner pericardial skirt	SAPIEN 3 prosthesis, consisting of a balloon-expandable cobalt-chromium frame accommodating a tri-leaflet valve manufactured from bovine pericardial tissue	**Page 4: 'C**ombination of two VARC-2- derived endpoints (early safety and clinical efficacy) at 30 days, as follows: all-cause death, any stroke, lifethreatening or disabling bleeding, major vascular complications, coronary artery obstruction requiring intervention, acute kidney injury (stage 2 or higher), rehospitalization for valve-related symptoms or congestive heart failure'	30 days	372	367	87	60	9	3	1.43	2.96	0.33	-0.10	0.76	0.0020	Risk difference	Yes	Yes	https://clinicaltrials.gov/ct2/show/NCT03011346
15	Araújo *et al.* 2020	BRAzil magnesium (BRAMAG) trial: a double-masked randomized clinical trial of oral magnesium supplementation in pregnancy	BMC Pregnancy and Childbirth	10.1186/s12884-020-02935-7	Daily magnesium citrate capsule (300 mg elemental magnesium citrate per capsule)	Placebo	**Page 3:** 'Composite outcome comprised preterm birth < 37 weeks' gestation, stillbirth > 20 weeks, neonatal death or NICU admission < 28 days after birth, or small for gestational age birthweight < 3rd percentile.'	28 weeks	449	462	75	76	4	10	1.02	0.41	-0.02	-0.35	0.32	0.0000	Odds Ratio	Yes	Yes	https://clinicaltrials.gov/ct2/show/NCT02032186
16	Gershlick *et al* . 2019	Long-Term Follow-Up of Complete Versus Lesion-Only Revascularization in STEMI and Multivessel Disease	Journal of the American College of Cardiology	10.1016/j.jacc.2019.10.033	Complete revascularization in patients with ST-segment elevation myocardial infarction	Infarct-related artery–only PCI	**Page 3084:** 'Composite of all-cause death, myocardial infarction [MI], ischemia-driven revascularization [IDR], and heart failure).'	5.6 years	150	146	36	55	9	15	0.64	0.58	0.84	-0.28	1.95	0.7759	Hazard Ratio	Yes	Yes	https://www.isrctn.com/ISRCTN70913605
17	Tajstra *et al.* 2020	Remote Supervision to Decrease Hospitalization Rate (RESULT) study in patients with implanted cardioverterdefibrillator	Europace	10.1093/europace/euaa072	Remote monitoring care (RC) of cardiac implantable electronic devices	Standard care	**Page 3:** 'Composite of all-cause death or hospitalization due to cardiovascular reasons.'	12 months	299	301	118	146	18	18	0.81	1.01	-30.94	-2978.49	2916.62	0.9831	Hazard Ratio	Yes	Yes	https://www.clinicaltrials.gov/ct2/show/NCT02409225
18	Lacasse *et al* . 2020	Randomized Trial of Nocturnal Oxygen in Chronic Obstructive Pulmonary Disease	The New England Journal of Medicine	10.1056/NEJMoa2013219	Nocturnal Oxygen in Chronic Obstructive Pulmonary Disease	Ambient air from a sham concentrator	**Page 1131**: 'Composite of death from any cause or a requirement for long-term oxygen therapy.'	4 years	123	120	57	61	31	30	0.91	1.01	-11.43	-644.03	621.18	0.9693	Hazard Ratio	Yes	Yes	https://clinicaltrials.gov/ct2/show/NCT01044628
19	Koch *et al.* 2020	Effect of red blood cell storage duration on major postoperative complications in cardiac surgery: A randomized trial	The Journal of Thoracic and Cardiovascular Surgery	10.1016/j.jtcvs.2019.09.165	Transfusion of RBCs stored for > or = 14 days (younger units) intraoperatively and throughout the postoperative hospitalization.	Transfusion of RBCs stored for > or = 20 days (older units) intraoperatively and throughout the postoperative hospitalization.	**Page 1506:** 'Mortality or multisystem organ failure, cardiac events (ventricular tachycardia, fibrillation, or asystole; atrial fibrillation), pulmonary events (pneumonia, prolonged postoperative ventilation, pulmonary embolus), neurologic events (stroke, coma), renal failure, infection (deep sternal wound infection, sepsis), gastrointestinal events (ischemia, infarction), any reoperation (for bleeding, tamponade, cardiac dysfunction), and vascular events (dissection, limb [illegible]	-	1921	1914	553	594	15	23	0.93	0.65	0.17	-0.14	0.49	0.0000	Odds Ratio	Yes	Yes	https://clinicaltrials.gov/ct2/show/NCT00458783
20	Zhang *et al* . 2019	An abluminal biodegradable polymer sirolimus-eluting stent versus a durable polymer everolimus-eluting stent in patients undergoing coronary revascularization: 3-year clinical outcomes of a randomized noninferiority trialasystole	Scientific Reports	10.1038/s41598-019-54964-8	Cordimax (Rientech Medical, Shandong, China) is a novel biodegradable polymer sirolimus-eluting stent with an asymmetric coating, eluting the drug solely to the abluminal surface in patients undergoing coronary revascularization	Durable polymer everolimus-eluting stent in patients undergoing coronary revascularization	**Page 1:** 'The primary efficacy endpoint was a target-lesion failure (TLF) at 1 year and the primary safety endpoint was a composite of death or myocardial infarction (MI).'	3 years	2460	1237	39	19	9	8	1.03	0.57	-0.06	-1.07	0.96	0.0407	Risk Relative	Yes	Yes	https://clinicaltrials.gov/ct2/show/NCT03185221
21	Kim *et al.* 2020	Durable Polymer Versus Biodegradable Polymer Drug-Eluting Stents After Percutaneous Coronary Intervention in Patients with Acute Coronary Syndrome: The HOST-REDUCE-POLYTECH-ACS Trial	Circulation	10.1161/CIRCULATIONAHA.120.051700	Durable Polymer Stents in patients with acute coronary syndrome (ACS) receiving percutaneous coronary intervention.	Biodegradable Polymer Drug-Eluting Stents in patients with acute coronary syndrome (ACS) receiving percutaneous coronary intervention.	**Page 6:** 'Composite of all-cause death, non-fatal myocardial 12 mothsinfarction (MI).'	12 months	1713	1700	87	106	43	50	0.81	0.85	1.29	-1.15	3.74	0.8134	Hazard Ratio	Yes	Yes	https://clinicaltrials.gov/ct2/show/NCT02193971
22	Shiomi *et al* . 2019	7-Year Outcomes of a Randomized Trial Comparing the First-Generation Sirolimus-Eluting Stent Versus the New-Generation Everolimus-Eluting Stent	JACC: Cardiovascular Interventions	10.1016/j.jcin.2019.01.234	New-Generation Everolimus-Eluting Stent	First-Generation Sirolimus-Eluting Stent	**Page 629:** 'Composite of death or MI'.	7 years	1335	1332	263	300	214	234	0.87	0.91	1.46	-0.06	2.99	0.5526	Hazard Ratio	Yes	Yes	https://clinicaltrials.gov/ct2/show/NCT01035450
23	Nguyen *et al.* 2019	Effect of Physical Activity Coaching on Acute Care and Survival Among Patients With Chronic Obstructive Pulmonary Disease	JAMA Network Open	10.1001/jamanetworkopen.2019.9657	Physical Activity Coaching included collaborative monitoring of PA step counts, semiautomated step goal recommendations, individualized reinforcement, and peer/family support, with built-in flexibility to [illegible]	Standard care	**Page 4:** 'Composite binary measure of any all-cause hospitalizations, observation stays, emergency department visits, and death.'	12 months	1358	1349	883	864	117	117	1.02	0.99	-2.27	-88.24	83.70	0.9405	Odds Ratio	Yes	Yes	https://clinicaltrials.gov/ct2/show/NCT02478359
24	Pels *et al* . 2020	Maternal Sildenafil vs Placebo in PregnantWomen With Severe Early-Onset Fetal Growth Restriction A Randomized Clinical Trial	JAMA Network Open	10.1001/jamanetworkopen.2020.5323	Sildenafil 25mg taken orally 3 times daily in PregnantWomen With Severe Early-Onset Fetal Growth Restriction	Placebo	**Page 1:** 'Composite of perinatal mortality or major neonatal morbidity until hospital discharge.'	Neonatal period as the time until hospital discharge	108	108	65	58	44	40	1.12	1.10	1.20	-1.86	4.25	0.9003	Risk Relative	Yes	Yes	https://clinicaltrials.gov/ct2/show/NCT02277132
25	Schepers *et al.* 2020	Urgent endoscopic retrograde cholangiopancreatography with sphincterotomy versus conservative treatment in predicted severe acute gallstone pancreatitis (APEC): a multicentre randomised controlled trial	The Lancet	10.1016/S0140-6736(20)30539-0	Urgent endoscopic retrograde cholangiopancreatography (ERCP) with biliary sphincterotomy	Conservative treatment	**Page 169:** 'Composite of major complications or mortality occurring within 6 months after randomisation.'	6 months	118	114	45	50	8	10	0.87	0.77	0.54	-1.31	2.39	0.6280	Risk Relative	Yes	Yes	https://www.isrctn.com/ISRCTN15545919
26	Makkar *et al* . 2020	Self-expanding intra-annular versus commercially available transcatheter heart valves in high and extreme risk patients with severe aortic stenosis (PORTICO IDE): a randomised, controlled, non-inferiority trial	The Lancet	10.1016/S0140-6736(20)31358-1	The Portico valve is composed of three intra-annular, bovine pericardial tissue valve leaflets mounted in a radiopaque, non-flared, self-expanding nitinol frame with a porcine pericardial valve sealing cuff in high and extreme risk patients with severe aortic stenosis.	Commercially available transcatheter heart valves in high and extreme risk patients with severe aortic stenosis	**Page 4:** 'The primary safety endpoint was a non-hierarchical composite of all-cause mortality, disabling stroke, lifethreatening bleeding requiring blood transfusion, acute kidney injury requiring dialysis, or major vascular complications at 30 days after randomisation.'	30 days	381	369	52	35	13	7	1.44	1.80	0.62	-0.28	1.52	0.4063	Absolute Difference	Yes	Yes	https://clinicaltrials.gov/ct2/show/NCT02000115
26	Makkar *et al* . 2020	Self-expanding intra-annular versus commercially available transcatheter heart valves in high and extreme risk patients with severe aortic stenosis (PORTICO IDE): a randomised, controlled, non-inferiority trial	The Lancet	10.1016/S0140-6736(20)31358-1	The Portico valve is composed of three intra-annular, bovine pericardial tissue valve leaflets mounted in a radiopaque, non-flared, self-expanding nitinol frame with a porcine pericardial valve sealing cuff in high and extreme risk patients with severe aortic stenosis.	Commercially available transcatheter heart valves in high and extreme risk patients with severe aortic stenosis	**Page 4:** 'The primary efficacy endpoint was a non-hierarchical composite of allcause mortality or disabling stroke at 1 year after randomisation.'	1 year	381	369	55	48	53	43	1.11	1.19	0.59	-0.32	1.50	0.3760	Absolute Difference	Yes	Yes	https://clinicaltrials.gov/ct2/show/NCT02000115
27	Smits *et al.* 20	Fractional flow reserve-guided multivessel angioplasty in myocardial infarction: three-year follow-up with cost benefit analysis of the Compare-Acute trial	EuroIntervention	10.4244/EIJ-D-20-00012	Fractional flow reserve (FFR)-guided acute complete revascularisation	infarct-related artery (IRA)-only treatment (Culprit)	**Page 227**: 'All-cause mortality, non-fatal MI, any revascularisation and cerebrovascular events (MACCE) at one year.'	36 monts	295	590	9	21	46	178	0.52	0.86	4.28	-16.41	24.97	0.7559	Hazard Ratio	Yes	Yes	https://clinicaltrials.gov/ct2/show/NCT01399736
28	Weber *et al.* 2019	Implementation of a Brazilian Cardioprotective Nutritional (BALANCE) Program for improvement on quality of diet and secondary prevention of cardiovascular events: A randomized, multicenter trial	American Heart Journal	10.1016/j.ahj.2019.06.010. Epub 2019 Jun 21	Nutritional program: BALANCE	Conventional nutrition advice	**Page 190** 'Composite of all-cause mortality, cardiovascular death, cardiac arrest, acutemyocardial infarction, stroke, myocardial revasculariza-tion, amputation for peripheral arterial disease, orhospitalization for unstable angina.'	3.5 years	1266	1268	236	207	98	97	1.14	1.01	11.21	-235.53	257.95	0.9353	Hazard Ratio	Yes	Yes	https://clinicaltrials.gov/ct2/show/NCT01620398
29	Matsumura-Nakano *et al.* 2019	Open-Label Randomized Trial Comparing Oral Anticoagulation With and Without Single Antiplatelet Therapy in Patients With Atrial Fibrillation and Stable Coronary Artery Disease Beyond 1 Year After Coronary Stent Implantation	Circulation	10.1161/CIRCULATIONAHA.118.036768	Oral anticoagulation (OAC) alone	Oral anticoagulation (OAC) and Antiplatelet Therapy	**Page 606**: ' Composite of all-cause death, myocardial infarction (MI), stroke, or systemic embolism.'	2.5 years	347	346	54	47	40	31	1.15	1.29	0.54	-0.32	1.40	0.2953	Hazard Ratio	Yes	Yes	https://www.clinicaltrials.gov/ct2/show/NCT01962545
30	Behr *et al.* 2020	Efficacy and safety of sildenafil added to pirfenidone in patients with advanced idiopathic pulmonary fibrosis and risk of pulmonary hypertension: a double-blind, randomised, placebo-controlled, phase 2b trial	The Lancet Respiratory Medicine	10.1016/S2213-2600(20)30356-8	Sildenafil and pirfenidone	Placebo and pirfenidone	**Page 4:** 'Relevant decline in 6-min walk distance from baseline, respiratory-related non-elective admission to hospital, or all-cause mortality.'	52 weeks	88	89	64	62	15	18	1.04	0.84	-0.25	-1.88	1.37	0.1308	Hazard Ratio	Yes	Yes	https://clinicaltrials.gov/ct2/show/NCT02951429
31	Onland *et al.* 2019	Effect of Hydrocortisone Therapy Initiated 7 to 14 Days After Birth on Mortality or Bronchopulmonary Dysplasia Among Very Preterm Infants Receiving Mechanical Ventilation	JAMA	10.1001/jama.2018.21443	22-day course of systemic hydrocortisone (cumulative dose, 72.5mg/kg)	Placebo	**Page 356:** 'Composite of death or bronchopulmonary dysplasia (BPD) at 36 weeks' postmenstrual age.'	36 weeks	182	190	128	140	28	45	0.95	0.65	0.11	-0.17	0.39	0.0000	Odds Ratio	Yes	Yes	https://www.trialregister.nl/trial/2640
32	Lee *et al.* 2019	Randomized Trial Evaluating Percutaneous Coronary Intervention for the Treatment of Chronic Total Occlusion	Circulation	10.1161/CIRCULATIONAHA.118.031313	Coronary intervention (PCI)in coronary vessels with chronic total occlusion (CTO) CTO-PCI	No CTO-PCI	**Page 1676**: 'Composite of death from any cause, MI, stroke, or any revascularization.'	36 monts	417	398	93	89	15	21	1.00	0.68	0.01	-0.66	0.67	0.0034	Hazard Ratio	Yes	Yes	https://www.clinicaltrials.gov/ct2/show/NCT01078051
33	Ulrich *et al.* 2020	Treating COVID-19 With Hydroxychloroquine (TEACH): A Multicenter, Double-Blind Randomized Controlled Trial in Hospitalized Patients	Open Forum Infectious Diseases	10.1093/ofid/ofaa446	Hydroxychloroquine (HCQ)	Placebo	**Page 3:** 'The proportion of subjects meeting a severe COVID-19 progression composite end point (death, ICU admission, mechanical ventilation, ECMO, and/or vasopressor use) at day 14.'	14 days	67	61	11	6	3	5	1.67	0.55	-0.85	-4.04	2.35	0.2568		Yes	Yes	https://clinicaltrials.gov/ct2/show/NCT04369742
34	Chotechuang *et al.* 2019	Cardiovascular outcomes of early versus delayed coronary intervention in low to intermediate-risk patients with STEMI in Thailand: a randomised trial	Heart Asia	10.1136/heartasia-2019-011201	Early coronary intervention	Delayed coronary intervention	**Page 2**: 'Composite of all-cause death, rehospitalisation with acute coronary syndrome, rehospitalisation with heart failure (HF) and stroke.'	6 months	81	81	13	5	4	3	2.60	1.33	3.32	-11.51	18.15	0.7590	Hazard Ratio	Yes	Yes	https://clinicaltrials.gov/ct2/show/NCT02131103
35	Rasmussen *et al.* 2019	Comparison of Acute Versus Subacute Coronary Angiography in Patients With NON-ST-Elevation Myocardial Infarction (from the NONSTEMI Trial)	The American Journal of Cardiology	10.1016/j.amjcard.2019.06.007	Acute coronary angiography (CAG)	Medical therapy and subacute CAG.	**Page 826:** 'Composite of all-cause mortality, reinfarction or readmission with CHF.'	1 year	247	253	25	29	14	14	0.88	1.02	-5.18	-176.08	165.72	0.9435		Yes	Yes	https://clinicaltrials.gov/ct2/show/NCT01638806
36	Chen *et al.* 2018	Post-discharge short message service improves shortterm clinical outcome and self-care behaviour in chronic heart failure	ESC Heart Failure	10.1002/ehf2.12380	Short message service (SMS)	Usual care	**Page 3**: 'Composite of all-cause hospitalization or death.'	180 days	252	260	104	131	26	28	0.82	0.96	4.66	-48.69	58.00	0.8931	Hazard Ratio	Yes	Yes	http://www.chictr.org.cn/enindex.aspx
36	Chen *et al.* 2019	Post-discharge short message service improves shortterm clinical outcome and self-care behaviour in chronic heart failure	ESC Heart Failure	10.1002/ehf2.12380	Structured telephone support (STS)	Usual care	**Page 3**: 'Composite of all-cause hospitalization or death.'	180 days	255	260	93	131	22	28	0.72	0.80	1.46	-1.80	4.71	0.7829	Hazard Ratio	Yes	Yes	http://www.chictr.org.cn/enindex.aspx
36	Chen *et al.* 2019	Post-discharge short message service improves shortterm clinical outcome and self-care behaviour in chronic heart failure	ESC Heart Failure	10.1002/ehf2.12380	Short message service (SMS)	Structured telephone support (STS)	**Page 3**: 'Composite of all-cause hospitalization or death.'	180 days	252	255	104	93	26	22	1.13	1.20	0.69	-1.25	2.64	0.7557	Hazard Ratio	Yes	Yes	http://www.chictr.org.cn/enindex.aspx
37	Kufner *et al.* 2019	Ten-Year Clinical Outcomes From a Trial of Three Limus-Eluting Stents With Different Polymer Coatings in Patients With Coronary Artery Disease	Circulation	10.1161/CIRCULATIONAHA.118.038065	New-generation BP-SES	Early generation PP-SES	**Page 326:** 'Composite of death, myocardial infarction, or target lesion revascularization.'	10.6 years	1299	652	575	223	374	223	0.86	0.84	0.88	0.38	1.38	0.6467	Hazard Ratio	Yes	Yes	https://www.clinicaltrials.gov/ct2/show/NCT00598676
37	Kufner *et al.* 2019	Ten-Year Clinical Outcomes From a Trial of Three Limus-Eluting Stents With Different Polymer Coatings in Patients With Coronary Artery Disease	Circulation	10.1161/CIRCULATIONAHA.118.038065	New-PP-EES	Early generation PP-SES	**Page 326:** 'Composite of death, myocardial infarction, or target lesion revascularization.'	10.6 years	652	652	279	336	179	223	0.83	0.80	0.85	0.39	1.30	0.5059	Hazard Ratio	Yes	Yes	https://www.clinicaltrials.gov/ct2/show/NCT00598676
37	Kufner *et al.* 2019	Ten-Year Clinical Outcomes From a Trial of Three Limus-Eluting Stents With Different Polymer Coatings in Patients With Coronary Artery Disease	Circulation	10.1161/CIRCULATIONAHA.118.038065	New-generation BP-SES	New-PP-EES	**Page 326:** 'Composite of death, myocardial infarction, or target lesion revascularization.'	10.6 years	1299	652	575	279	374	179	1.03	1.05	0.71	-1.01	2.43	0.7423	Hazard Ratio	Yes	Yes	https://www.clinicaltrials.gov/ct2/show/NCT00598676
38	Hollemans *et al.* 2018	Superiority of Step-up Approach Vs Open Necrosectomy in Long-term Follow-up of Patients With Necrotizing Pancreatitis	Gastroenterology	10.1053/j.gastro.2018.10.045	Step-up approach	Primary open necrosectomy	**Page 3:** 'Composite of death or major complications (ie, new-onset multiple organ failure or systemic complications, enterocutaneous fistula or perforation of a visceral organ requiring intervention, or intra-abdominal bleeding requiring intervention at any time during follow-up.'	86 months	43	45	19	33	13	9	0.60	1.51	-1.23	-4.01	1.55	0.1166	Relative Risk	Yes	Yes	https://clinicaltrials.gov/ct2/show/NCT01266187

39	Lee *et al.* 2019	Invasive Versus Medical Management in Patients With Prior Coronary Artery Bypass Surgery With a Non-ST Segment Elevation Acute Coronary Syndrome A Pilot Randomized Controlled Trial	Circulation: Cardiovascular Interventions	10.1161/CIRCINTERVENTIONS.119.007830	Invasive management: native coronary and bypass graft angiography and coronary and graft revascularization with percutaneous coronary intervention and CABG.	Noninvasive management	**Page 5:** 'Composite of all-cause mortality, rehospitalization for refractory ischemia/angina, MI, or hospitalization for HF.'	744 days	31	29	17	16	5	3	0.99	1.56	-0.01	-1.06	1.03	0.0575	Hazard Ratio	Yes	Yes	https://clinicaltrials.gov/ct2/show/NCT01895751
40	Ryan *et al.* 2019	Perioperative haemodynamic therapy for major gastrointestinal surgery: the effect of a Bayesian approach to interpreting the findings of a randomised controlled trial	BMJ Open	10.1136/bmjopen-2018-024256	Patients received intravenous fluid and inotropes according to a cardiac output–guided hemodynamic therapy algorithm	Usual Care	**Page 2**: 'Composite of predefined 30-day moderate or major complications and mortality.'	30 days	368	366	134	158	12	11	0.84	1.08	-2.09	-23.29	19.11	0.7753	Relative Risk	Yes	Yes	https://www.isrctn.com/ISRCTN04386758
41	Castella *et al.* 2019	Thoracoscopic vs. catheter ablation for atrial fibrillation: long-term follow-up of the FAST randomized trial	European Society of Cardiology	10.1093/europace/euy325	Thoracoscopic ablation in patients with drug-refractory AF referred for further rhythm control	Catheter ablation	**Page 748**: 'Composite cumulative frequency of death, MI, or cerebrovascular event (transient ischaemic attack or ischaemic/haemorrhagic stroke.'	12 months	63	66	9	10	4	5	0.94	0.84	0.33	-3.29	3.96	0.7183	Hazard Ratio	Yes	Yes	https://www.clinicaltrials.gov/ct2/show/NCT00662701
42	Erlinge *et al.* 2019	Bivalirudin versus heparin monotherapy in non-ST-segment elevation myocardial infarction	European Heart Journal. Acute Cardiovascular Care	10.1177/2048872618805663	Bivalirudin	Heparin	**Page 497**: 'Composite of all-cause death, myocardial infarction or major bleeding.'	180 days	1503	1498	182	187	30	26	0.97	1.15	-0.22	-2.05	1.61	0.1916	Hazard Ratio	Yes	Yes	https://clinicaltrials.gov/ct2/show/NCT02311231
43	He *et al.* 2019	Percutaneous Drainage Versus Peritoneal Lavage for Pancreatic Ascites in Severe Acute Pancreatitis	Pancreas	10.1097/MPA.0000000000001251	Percutaneous drainage (PCD)	Peritoneal lavage (PL)	**Page 344**: 'Composite of mortality or major complications, which included new-onset multiple-organ failure and infected pancreatic necrosis (IPN).'	1 month	43	43	15	17	6	6	0.88	1.00	NC	NC	NC	NC	Relative Risk	No	Yes	http://www.chictr.org.cn/showprojen.aspx?proj=8041
44	Feig *et al.* 2020	Metformin in women with type 2 diabetes in pregnancy (MiTy): a multicentre, international, randomised, placebo- controlled trial	The Lancet Diabetes & Endocrinology	10.1016/S2213-8587(20)30310-7	Metformin and Insulin	Placebo and standard regimen of insulin	**Page 837**: 'Fetal and neonatal outcomes.'	20.9 weeks	253	249	94	95	13	14	0.97	0.91	0.29	-2.59	3.18	0.6320	Relative Risk	Yes	Yes	https://clinicaltrials.gov/ct2/show/NCT01353391
45	Wilson *et al.* 2020	Effect of Co-trimoxazole (Trimethoprim-Sulfamethoxazole) vs Placebo on Death, Lung Transplant, or Hospital Admission in Patients With Moderate and Severe Idiopathic Pulmonary Fibrosis The EME-TIPAC Randomized Clinical Trial	JAMA	10.1001/jama.2020.22960	Co-trimoxazole	Placebo	**Page 2283:** 'Time to death (all causes), lung transplant, or first nonelective hospital admission for any reason.'	12 months	169	172	84	80	24	18	1.07	1.36	0.22	-0.46	0.90	0.0240	Hazard Ratio	Yes	Yes	https://www.isrctn.com/ISRCTN17464641
46	Watanabe *et al.* 2020	Remote Management of Pacemaker Patients With Biennial In-Clinic Evaluation	Circulation: Arrhythmia and Electrophysiology	10.1161/CIRCEP.119.007734.	Exclusive remote follow-up (RFU)	Conventional follow-up	**Page 420:** 'Composite of death, stroke, or cardiovascular surgical procedure.'	2 years	636	638	61	65	46	41	0.94	1.13	-0.51	-4.87	3.85	0.4969		Yes	Yes	https://clinicaltrials.gov/ct2/show/NCT01523704
47	Karaye *et al.* 2020	Selenium supplementation in patients with peripartum cardiomyopathy: a proof-of-concept trial	BMC Cardiovascular Disorders	10.1186/s12872-020-01739-z	Oral Selenium (L-selenomethionine) and HF standard treatment	Nothing and HF standard treatment	**Page 3**: 'Composite of persistence of HF symptoms, unrecovered LVEF (less than 55%) or death from any cause.'	19 months	46	54	36	43	3	9	0.98	0.39	0.02	-0.20	0.23	0.0000	Hazard Ratio	Yes	Yes	https://clinicaltrials.gov/ct2/show/NCT03081949
48	Myles *et al.* 2019	Aspirin in coronary artery surgery: 1-year results of the Aspirin and Tranexamic Acid for Coronary Artery Surgery trial	The Journal of Thoracic and Cardiovascular Surgery	10.1016/j.jtcvs.2018.08.114	Aspirin	Placebo	**Page 634**: 'Death or disability, the latter was defined as a surviving patient with a modified Katz ADL score<8.'	12 months	1059	1068	42	36	35	26	1.18	1.36	0.53	-0.26	1.32	0.2467	Relative Risk	No	Yes	https://www.anzctr.org.au/Trial/Registration/TrialReview.aspx?ACTRN=12605000557639
49	Futier *et al.* 2020	Effect of Hydroxyethyl Starch vs Saline for Volume Replacement Therapy on Death or Postoperative Complications Among High-Risk Patients Undergoing Major Abdominal Surgery: The FLASH Randomized Clinical Trial	JAMA	10.1001/jama.2019.20833	Fluid containing either 6% Hydroxyethyl starches (HES) 130/0.4 diluted in 0.9% saline	0.9% saline alone	**Page 227:** 'Composite of death or preselected major postoperative complications, including acute kidney injury of stage 1 or higher according to the Kidney Disease: Improving Global Outcomes (KDIGO) criteria, acute respiratory failure requiring invasive or noninvasive mechanical ventilation, acute heart failure, major septic complications, and unplanned reoperation 14 days after surgery.'	14 days	408	409	139	125	12	6	1.11	2.00	0.16	-0.17	0.48	0.0000	Relative Risk	Yes	Yes	https://clinicaltrials.gov/ct2/show/NCT02502773
50	Kalstad *et al.* 2020	Effects of n-3 Fatty Acid Supplements in Elderly Patients After Myocardial Infarction: A Randomized, Controlled Trial	Circulation	10.1001/jama.2020.0103	1.8 g n-3 PUFA - 930 mg icosapentaenoic acid (EPA) + 660 mg docosahexaenoic acid (DHA)	Placebo (corn oil; 56% linoleic acid, 32% oleic acid, 10% palmitic acid).	**Page 530:** 'Composite of nonfatal MI, unscheduled revascularization, stroke, or all-cause death.'	2 years	513	514	108	102	28	28	1.06	1.00	30.35	-7849.96	7910.66	0.9942	Hazard Ratio	Yes	Yes	https://clinicaltrials.gov/ct2/show/NCT01841944
51	Steven *et al.* 2020	Effect of Vancomycin or Daptomycin With vs Without an Antistaphylococcal β-Lactam on Mortality, Bacteremia, Relapse, or Treatment Failure in Patients With MRSA Bacteremia A Randomized Clinical Trial	JAMA	10.1001/jama.2020.0103	Standard therapy (intravenous vancomycin or daptomycin) plus an antistaphylococcal β-lactam (intravenous flucloxacillin, cloxacillin, or cefazolin)	Standard therapy alone	**Page 529:** 'Composite measure assessed 90 days after randomization with 4 components: (1) all-cause mortality; (2) persistent bacteremia at study day 5; (3) microbiological relapse defined as a positive blood culture for MRSA at least 72 hours after a preceding negative culture; and (4) microbiological treatment failure defined as a positive sterile site culture for MRSA at least 14 days after randomization.'	90 days	176	180	59	68	35	28	0.89	1.28	-0.49	-2.32	1.35	0.1122	Risk difference	No	Yes	https://clinicaltrials.gov/ct2/show/NCT02365493
52	Yasuda *et al.* 2019	Antithrombotic Therapy for Atrial Fibrillation with Stable Coronary Disease	The New England Journal of Medicine	10.1056/NEJMoa1904143	Monotherapy with rivaroxaban (a non–vitamin K antagonist oral anticoagulant)	Combination therapy with rivaroxaban plus a single antiplatelet agent	**Page 1105:** 'Composite of stroke, systemic embolism, myocardial infarction, unstable angina requiring revascularization, or death from any cause.'	24.1 months	1118	1118	89	121	41	73	0.74	0.56	0.53	0.21	0.86	0.0048	Hazard Ratio	Yes	Yes	https://clinicaltrials.gov/ct2/show/NCT02642419
53	Park *et al.* 2020	Ten-Year Outcomes After Drug-Eluting Stents Versus Coronary Artery Bypass Grafting for Left Main Coronary Disease	Circulation	10.1161/CIRCULATIONAHA.120.046039	Percutaneous coronary intervention (PCI) with sirolimus-eluting stents	Coronary-artery bypass grafting (CABG)	**Page 1439:** 'Composite of major adverse cardiac and cerebrovascular events (ie, death from any cause, nonfatal MI, nonfatal stroke, or ischemia-driven target vessel revascularization).'	10 years	300	300	87	72	42	40	1.21	1.05	3.88	-24.74	32.50	0.8437	Hazard Ratio	Yes	Yes	https://clinicaltrials.gov/ct2/show/NCT00422968
54	Macdougall *et al.* 2019	Intravenous Iron in Patients Undergoing Maintenance Hemodialysis	The New England Journal of Medicine	10.1056/NEJMoa1810742	High-dose intravenous iron administered proactively	Regimen of low-dose intravenous iron administered reactively	**Page 449:** 'Composite of nonfatal myocardial infarction, nonfatal stroke, hospitalization for heart failure, or death from any cause, assessed in a time-to-first-event analysis.'	2.1 years	1093	1048	320	338	246	269	0.91	0.88	0.74	0.22	1.26	0.3199	Estimated Treatment Effect	Yes	Yes	https://www.clinicaltrialsregister.eu/ctr-search/search?query=2013-002267-25
55	Park *et al.* 2020	Additional postdilatation using noncompliant balloons after everolimus-eluting stent implantation: Results of the PRESS trial	Clinical Cardiology	10.1002/clc.23355	High-pressure postdilatation using a noncompliant balloon	Moderate-pressure dilatation using a stent balloon	**Page 608:** 'Occurrence of major adverse cardiac events, including all-cause death, MI, stent thrombosis, and target vessel revascularization (TVR) 2 years after the procedure.'	2 years	406	404	14	17	3	6	0.82	0.50	0.29	-0.58	1.15	0.1038		Yes	No	
56	Sekiziyivu *et al.* 2020	Task Shifting for Initiation and Monitoring of Antiretroviral Therapy for HIV-Infected Adults in Uganda: The SHARE Trial	Journal of Acquired Immune Deficiency Syndromes	10.1097/QAI.0000000000002567	Nurse-initiated and monitored ART (NIMART)	Clinician-initiated and monitored ART (CIMART)	**Page e72:** Composite end point of treatment-limiting events comprising all-cause mortality, virological failure (VF), treatment-limiting ART toxic effects, and LTFU at 12 months.'	12 months	875	885	284	314	11	8	0.91	1.39	-0.27	-1.16	0.62	0.0051	Risk Difference	Yes	No	https://clinicaltrials.gov/ct2/show/NCT02417636
57	Willems *et al.* 2020	Preventive or Deferred Ablation of Ventricular Tachycardia in Patients With Ischemic Cardiomyopathy and Implantable Defibrillator (BERLIN VT): A Multicenter Randomized Trial	Circulation	10.1161/CIRCULATIONAHA.119.043400	Receive catheter ablation for VT either before ICD implantation (preventive ablation strategy, within 2 weeks of enrollment)	After the third appropriate ICD shock for VT (deferred ablation strategy)	**Page 1059:** 'Composite of all-cause death and unplanned hospitalization (at least 1 overnight stay) for either symptomatic ventricular tachyarrhythmia or worsening heart failure.'	396 days	77	86	25	23	6	2	1.21	3.35	0.16	-0.22	0.55	0.0000	Hazard Ratio	Yes	Yes	https://www.clinicaltrials.gov/ct2/show/NCT02501005
58	Mark *et al.* 2019	Transcatheter Aortic-Valve Replacement with a Balloon-Expandable Valve in Low-Risk Patients	The New England Journal of Medicine	10.1056/NEJMoa1814052	Transcatheter aortic-valve replacement (TAVR) with transfemoral placement of the balloon expandable SAPIEN 3 system	Surgical aortic-valve replacement with a commercially available bioprosthetic valve	**Page 1696**: 'Composite of death from any cause, stroke, or rehospitalization at 1 year after the procedure.'	1 year	503	497	42	68	5	11	0.61	0.45	0.62	-0.16	1.40	0.3350	Risk difference	Yes	Yes	https://clinicaltrials.gov/ct2/show/NCT02675114
59	Thiele *et al.* 2020	General Versus Local Anesthesia With Conscious Sedation in Transcatheter Aortic Valve Implantation: The Randomized SOLVE-TAVI Trial	Circulation	10.1161/CIRCULATIONAHA.120.046451	Local anesthesia with conscious sedation (CS)	General anesthesia (GA)	**Page 1440:** 'Composite of all-cause mortality, stroke, myocardial infarction, infection requiring antibiotic treatment, and acute kidney injury at 30-days follow-up.'	30 days	222	225	59	58	7	5	1.03	1.42	0.09	-0.77	0.94	0.0367	Rate difference	Yes	Yes	https://clinicaltrials.gov/ct2/show/NCT02737150
60	Ho *et al.* 2019	A Multicenter Trial of Vena Cava Filters in Severely Injured Patients	The New England Journal of Medicine	10.1056/NEJMoa1806515	Retrievable filter (vena cava filter group)	No filter (control group)	**Page 330:** 'Composite of symptomatic pulmonary embolism (segmental pulmonary embolism on CT pulmonary angiography, confirmed by na independent consultant radiologist or by postmortem examination) or death from any cause at 90 days after enrollment.'	90 days	112	118	17	17	16	11	0.97	1.41	-0.10	-2.10	1.91	0.2834	Hazard Ratio	Yes	Yes	https://www.anzctr.org.au/Trial/Registration/TrialReview.aspx?ACTRN=12614000963628
61	Thyregod *et al.* 2019	Five-Year Clinical and Echocardiographic Outcomes From the NOTION Randomized Clinical Trial in Patients at Lower Surgical Risk	Circulation	10.1161/CIRCULATIONAHA.118.036606	Transcatheter aortic valve replacement (TAVR)	Surgical aortic valve replacement (SAVR)	**Page 2716:** 'Clinical outcomes of all-cause mortality, cardiovascular mortality, stroke, and MI.'	5 years	145	135	55	49	40	39	1.05	0.95	-0.95	-14.64	12.73	0.7795	Hazard Ratio	Yes	Yes	https://clinicaltrials.gov/ct2/show/NCT01057173
62	Pejovic *et al.* 2020	A Randomized Trial of Laryngeal Mask Airway in Neonatal Resuscitation	The New England Journal of Medicine	10.1056/NEJMoa2005333	Laryngeal mask airway	Face mask	**Page 2141:** 'Composite of death within 7 days or admission to the NICU with moderate-to-severe hypoxic–ischemic encephalopathy (Thompson score, ≥11) at day 1 to 5 during hospitalization.'	7 days	566	597	154	144	122	109	1.13	1.18	0.73	0.10	1.35	0.3915	Relative Risk	Yes	Yes	https://clinicaltrials.gov/ct2/show/NCT03133572
63	Thiele *et al.* 2020	Comparison of newer generation self-expandable vs. balloon-expandable valves in transcatheter aortic valve implantation: the randomized SOLVE-TAVI trial	European Heart Journal	10.1093/eurheartj/ehaa167	Self-expandable valve (SEV)	Balloon-expandable valve (BEV)	**Page 1892:** 'Composite of all-cause mortality, stroke, moderate or severe PVL, and permanent pacemaker implantation at 30-day follow-up.'	30 days	225	222	62	56	7	5	1.09	1.38	0.27	-0.88	1.43	0.2182	Rate difference	Yes	Yes	https://clinicaltrials.gov/ct2/show/NCT02737150
64	Nguye *et al.* 2020	Low-tidal volume mechanical ventilation against no ventilation during cardiopulmonary bypass in heart surgery (MECANO): a randomized controlled trial	Chest	10.1016/j.chest.2020.10.082	The ventilation group received 5 breaths/min with a tidal volume of3 mL/kg and PEEP of 5 cm H2O	The no ventilation group received no ventilation during cardiopulmonary bypass (CPB)	**Page 1844:** 'Composite of overall death; early respiratory failure, defined as PAO2to FIO2ratio of<200 during the first day of ICU care; advanced respiratory support, defined as apatient requiring either noninvasive ventilation, mechanicalventilation, or high-flow oxygen, at 2 days after arrival in the ICU; and hospital or ventilator-acquired pneumonia as defined by the Centers for Disease Control and Prevention.'	Until hospital discharge	756	745	112	113	9	13	0.83	0.68	0.49	-0.59	1.57	0.3528	Odds Ratio	Yes	Yes	https://clinicaltrials.gov/ct2/show/NCT03098524
65	Dangas *et al.* 2020	A Controlled Trial of Rivaroxaban after Transcatheter Aortic-Valve Replacement	The New England Journal of Medicine	10.1056/NEJMoa1911425	The anticoagulant group was assigned to receive rivaroxaban at a dose of 10 mg daily plus aspirin at a dose of 75 to 100 mg daily for 3 months, followed by rivaroxaban monotherapy (10 mg daily).	The antiplatelet group was assigned to receive aspirin at a dose of 75 to 100 mg daily plus clopidogrel at a dose of 75 mg daily for 3 months	**Page 122:** 'Composite of death from any cause or thromboembolic events, including any stroke, myocardial infarction, symptomatic valve thrombosis, systemic embolism (not involving the central nervous system), deep-vein thrombosis, or pulmonary embolism.'	17 months	826	818	105	78	64	38	1.33	1.67	0.56	0.18	0.95	0.0251	Risk Difference	Yes	Yes	https://clinicaltrials.gov/ct2/show/NCT02556203
66	Myles *et al.* 2019	Tranexamic acid in coronary artery surgery: One-year results of the Aspirin and Tranexamic Acid for Coronary Artery Surgery (ATACAS) trial	Journal of Thoracic and Cardiovascular Surgery	10.1016/j.jtcvs.2018.09.113	Tranexamic acid, 50 to 100 mg per kg	0.9% saline (placebo)	**Page 647:** 'Composite of death or disability; the latter was defined as a surviving patient with a modified Katz ADL score of less than 8.'	1 year	2329	2333	84	100	68	78	0.84	0.87	1.27	-0.22	2.77	0.7196	Relative Risk	No	Yes	https://www.anzctr.org.au/Trial/Registration/TrialReview.aspx?ACTRN=12605000557639
67	Vardeny *et al.* 2020	Effect of High-Dose Trivalent vs Standard-Dose Quadrivalent Influenza Vaccine on Mortality or Cardiopulmonary Hospitalization in Patients With High-risk Cardiovascular Disease: A Randomized Clinical Trial	JAMA	10.1001/jama.2020.23649	High-dose trivalent inactivated influenza vaccine (containing 60 µg of hemagglutinin per strain)	Standard-dose quadrivalent inactivated influenza vaccine (containing 15 µg of hemagglutinin per strain)	**Page 41:** 'Composite of all-cause death or hospitalization for cardiovascular or pulmonary causes during each enrolling influenza season, with censoring in the first 2 weeks after vaccination and after July 31 of the respective season.'	8.5 months	2630	2630	975	924	92	78	1.06	1.18	0.33	-0.31	0.96	0.0386	Hazard Ratio	No	Yes	https://clinicaltrials.gov/ct2/show/NCT02787044
68	Kojima *et al.* 2019	Febuxostat for Cerebral and CaRdiorenovascular Events PrEvEntion StuDy	European Heart Journal	10.1093/eurheartj/ehz119	Febuxostat preparation: (i) the starting febuxostat dose was 10mg/day; (ii) at week 4, the dose was increased to 20mg/day; (iii) at week 8, the dose was increased to the target dose of 40mg/day	Administration of 100mg of oral allopurinol	**Page 1780:** 'Fatal and non-fatal cerebral, cardiovascular and renal events, and death other than cerebral or cardiorenal vascular disease during the study period.'	36 months	537	533	125	153	10	12	0.81	0.83	1.10	-3.58	5.79	0.9652	Hazard Ratio	Yes	Yes	https://clinicaltrials.gov/ct2/show/NCT01984749
69	Hueb *et al.* 2019	Ten-Year Follow-Up of Off-Pump and On-Pump Multivessel Coronary Artery Bypass Grafting: MASS III	Angiology	10.1177/0003319718804402	Off-pump coronary artery bypass (OPCAB)	On-pump coronary artery bypass (ONCAB)	**Page 3:** 'Combined end points were freedom from overall mortality, stroke, myocardial infarction (MI), and additional revascularization.'	10 years	156	155	55	49	26	22	1.12	1.17	0.68	-1.19	2.55	0.7370	Hazard Ratio	Yes	Yes	https://www.isrctn.com/ISRCTN59539154
70	Tong *et al.* 2020	Colchicine in Patients With Acute Coronary Syndrome: The Australian COPS Randomized Clinical Trial	Circulation	10.1161/CIRCULATIONAHA.120.050771	Received 0.5 mg oral colchicine twice daily for the first month, followed by 0.5 mg daily for 11 months	Received placebo tablets	**Page 1892:** 'Composite of death from any cause, ACS (STEMI/non-STEMI/unstable angina), ischemiadriven urgent revascularization, and noncardioembolic ischemic stroke.'	371 days	396	399	24	38	8	1	0.64	8.06	-0.22	-0.57	0.14	0.0000	Hazard Ratio	Yes	Yes	https://www.anzctr.org.au/Trial/Registration/TrialReview.aspx?id=368973
71	Jain *et al.* 2019	Goat lung surfactant for treatment of respiratory distress syndrome among preterm neonates: a multi-site randomized non-inferiority trial	Journal of Perinatology	10.1038/s41372-019-0472-0	Goat lung surfactant extract (GLSE)	Standard bovine lung surfactant (beractant)	**Page 5:** 'Composite of death or bronchopulmonary dysplasia (BPD) defined physiologically at 36 weeks PMA as requirement of supplemental oxygen of 30% or more or the need for positive-pressure support to achieve oxygen saturation greater than 90%.'	36 months	52	46	26	18	21	14	1.28	1.33	0.87	-0.11	1.84	0.7882	Odds Ratio and Risk difference	Yes	Yes	https://clinicaltrials.gov/ct2/show/NCT02774044
72	Hahn *et al.* 2019	Effect of P2Y12 Inhibitor Monotherapy vs Dual Antiplatelet Therapy on Cardiovascular Events in Patients Undergoing Percutaneous Coronary Intervention: The SMART-CHOICE Randomized Clinical Trial	JAMA	10.1001/jama.2019.8146	P2Y12 inhibitor monotherapy group (aspirin plus a P2Y12 inhibitor for 3months and thereafter a P2Y12 inhibitor alone)	DAPT group (aspirin plus a P2Y12 inhibitor for at least 12 months)	**Page 2430:** 'Composite of all-cause death, myocardial infarction, or stroke at 12 months after the index procedure.'	12 months	1495	1498	42	36	21	18	1.17	1.17	1.00	-1.85	3.85	1.0000	Risk Difference	Yes	Yes	https://clinicaltrials.gov/ct2/show/NCT02079194
73	Schüpke *et al.* 2019	Ticagrelor or Prasugrel in Patients with Acute Coronary Syndromes	The New England Journal of Medicine	10.1056/NEJMoa1908973	Ticagrelor (dose of 180 mg and continued at a maintenance dose of 90 mg twice daily)	Prasugrel (dose of 60 mg and continued at a maintenance dose of 10 mg once per day)	**Page 1526:** 'Composite of death, myocardial infarction, or stroke at 1 year after randomization.'	1 year	2012	2006	184	137	90	73	1.34	1.23	1.41	-0.12	2.95	0.5968	Hazard Ratio	Yes	Yes	https://www.clinicaltrials.gov/ct2/show/NCT01944800
74	Prabhakaran *et al.* 2020	Yoga-Based Cardiac Rehabilitation After Acute Myocardial Infarction	Journal of the American College of Cardiology	10.1016/j.jacc.2020.01.050	Yoga-CaRe program (13 direct contact sessions spread over 12 weeks)	Standard care arm (3 sessions of educational advice with the help of a leaflet)	**Page 1553:** 'Composite of death from any cause, nonfatal myocardial infarction, nonfatal stroke, or emergency cardiovascular hospitalization.'	22 months	1970	1989	131	146	77	77	0.91	1.01	-10.29	-360.21	339.62	0.9496	Hazard Ratio	Yes	Yes	http://www.ctri.nic.in/Clinicaltrials/pmaindet2.php?trialid=3992
75	Zenati *et al.* 2019	Randomized Trial of Endoscopic or Open Vein-Graft Harvesting for Coronary-Artery Bypass	The New England Journal of Medicine	10.1056/NEJMoa1812390	Open vein-graft harvesting	Endoscopic vein-graft harvesting	**Page 134:** 'Composite of death from any cause, nonfatal myocardial infarction, or repeat revascularization) in a time-to-event analysis over the active follow-up period of the trial.'	2.8 years	574	576	89	80	46	37	1.12	1.25	0.50	-0.44	1.44	0.2961	Hazard Ratio	Yes	Yes	https://www.clinicaltrials.gov/ct2/show/NCT01850082
76	Johnston *et al.* 2020	Ticagrelor and Aspirin or Aspirin Alone in Acute Ischemic Stroke or TIA	The New England Journal of Medicine	10.1056/NEJMoa1916870	Ticagrelor (180-mg loading dose followed by 90 mg twice daily) plus aspirin (300 to 325 mg on the first day followed by 75 to 100 mg daily)	Placebo plus aspirin	**Page 209:** 'Composite of stroke or death in a time-to-first-event analysis from randomization through 30 days of follow-up.'	30 days	5523	5493	303	362	36	27	0.83	1.33	-0.65	-2.04	0.74	0.0204	Hazard Ratio	Yes	Yes	https://clinicaltrials.gov/ct2/show/NCT03354429
77	Blanc *et al* . 2020	Systematic or Test-Guided Treatment for Tuberculosis in HIV-Infected Adults	The New England Journal of Medicine	10.1056/NEJMoa1910708	The patients assigned to the systematic-treatment group underwent chest radiography but no other tuberculosis test, even if they reported tuberculosis symptoms	The patients assigned to the systematic-treatment group underwent chest radiography but no other tuberculosis test, even if they reported tuberculosis symptoms	**Page 2399:** 'Composite of death from any cause or invasive bacterial disease within 24 weeks.'	24 weeks	527	523	46	44	36	33	1.04	1.08	0.46	-2.52	3.45	0.7248	Hazard Ratio	Yes	Yes	https://clinicaltrials.gov/ct2/show/NCT02057796
78	Juul *et al* . 2020	A Randomized Trial of Erythropoietin for Neuroprotection in Preterm Infants	The New England Journal of Medicine	10.1056/nejmoa1907423	Erythropoietin - administered intravenously at a dose of 1000 U per kilogram of body weight every 48 hours for a total of six doses, followed by a maintenance dose of 400 U	Placebo - administered as intravenous saline followed by sham injections	**Page 235:** 'Composite of death or severe neurodevelopmental impairment at 22 to 26 months of postmenstrual age.'	22 to 26 months	477	464	97	94	63	50	1.00	1.23	0.02	-1.20	1.24	0.1154	Relative Risk	Yes	Yes	https://clinicaltrials.gov/ct2/show/NCT01378273
79	Sessler *et al* . 2020	One-year Results of a Factorial Randomized Trial of Aspirin versus Placebo and Clonidine versus Placebo in Patients Having Noncardiac Surgery	Anesthesiology	10.1097/aln.0000000000003158	200 mg of aspirin just before surgery	Placebo	**Page 694:** 'The primary outcome was a composite of death or nonfatal myocardial infarction.'	1 year	4998	5012	586	589	304	312	1.00	0.98	0.10	-4.10	4.30	0.6743	Hazard Ratio	Yes	Yes	https://clinicaltrials.gov/ct2/show/NCT01082874
79	Sessler *et al* . 2020	One-year Results of a Factorial Randomized Trial of Aspirin versus Placebo and Clonidine versus Placebo in Patients Having Noncardiac Surgery	Anesthesiology	10.1097/aln.0000000000003158	0.2 mg of oral clonidine	Placebo	**Page 694:** 'Composite of death or nonfatal myocardial infarction.'	1 year	5009	5001	608	567	313	303	1.07	1.03	2.21	-6.65	11.07	0.7890	Hazard Ratio	Yes	Yes	https://clinicaltrials.gov/ct2/show/NCT01082874
80	Popma *et al* . 2019	Transcatheter Aortic-Valve Replacement with a Self-Expanding Valve in Low-Risk Patients	The New England Journal of Medicine	10.1056/nejmoa1816885	Patients assigned to Transcatheter aortic-valve replacement (TAVR) were treated with one of three self-expanding, supraannular bioprostheses	Surgical aortic-valve replacement	**Page 1708:** 'Composite of death from any cause or disabling stroke at 24 months.'	12.2 months	734	734	21	34	18	22	0.62	0.82	2.40	-2.81	7.61	0.5979	Risk Difference	Yes	Yes	https://clinicaltrials.gov/ct2/show/NCT02701283
81	Iung *et al* . 2019	Percutaneous repair or medical treatment for secondary mitral regurgitation: outcomes at 2 years	European Journal of Heart Failure	10.1002/ejhf.1616	Percutaneous valve repair plus medical treatment	Medical treatment alone	**Page 1621:** 'Composite of all-causedeath or unplanned hospitalization for heart failure at 12months.'	24 months	152	152	97	102	53	52	0.95	1.02	-2.64	-50.61	45.33	0.8818	Hazard Raio	Yes	Yes	https://clinicaltrials.gov/ct2/show/NCT01920698

82	Stone *et al*. 2019	Five-Year Outcomes after PCI or CABG for Left Main Coronary Disease	The New England Journal of Medicine	10.1056/nejmoa1909406	Percutaneous coronary intervention (PCI)with fluoropolymer-based cobalt–chromium everolimus- eluting stents	Coronary-artery bypass grafting (CABG)	**Page 1821:** 'Composite of death from any cause, stroke, or myocardial infarction at 3 years.'	5 years	948	957	203	176	119	89	1.16	1.35	0.51	0.07	0.94	0.0257	Risk Difference and Odds Ratio	Yes	Yes	https://clinicaltrials.gov/ct2/show/NCT01205776
83	Kirpalani *et al*. 2020	Higher or Lower Hemoglobin Transfusion Thresholds for Preterm Infants	The New England Journal of Medicine	10.1056/NEJMoa2020248	Red-cell transfusions at higher hemoglobin thresholds until 36 weeks of postmenstrual age or discharge, whichever occurred first	Lower hemoglobin thresholds	**Page 2641:** 'Composite of death or neurodevelopmental impairment in infants at 22 to 26 months of age, corrected for prematurity.'	2 years	845	847	423	422	146	135	1.00	1.08	0.06	-1.06	1.18	0.1000	Relative Risk	No	Yes	https://clinicaltrials.gov/ct2/show/NCT01702805
84	Bangalore *et al*. 2020	Management of Coronary Disease in Patients with Advanced Kidney Disease	The New England Journal of Medicine	10.1056/nejmoa1915925	Coronary angiography and revascularization (if appropriate) added to medical therapy	Initial conservative strategy consisting of medical therapy alone	**Page 1610:** 'Composite of death or nonfatal myocardial infarction.'	2.2 years	388	389	123	129	94	98	0.96	0.96	1.15	-2.99	5.30	0.9426	Hazard Raio	Yes	Yes	https://clinicaltrials.gov/ct2/show/NCT01985360
85	Thrane *et al*. 2020	16-year follow-up of the Danish Acute Myocardial Infarction 2 (DANAMI-2) trial: primary percutaneous coronary intervention vs. fibrinolysis in ST-segment elevation myocardial infarction	European Heart Journal	10.1093/eurheartj/ehz595	Primary percutaneous coronary intervention (pPCI)	Fibrinolysis	**Page 850:** 'Composite endpoint of death or rehospitalization for MI in patients treated with pPCI compared to fibrinolysis.'	16 years	790	782	463	487	398	401	0.94	0.98	3.43	-11.93	18.80	0.7563	Hazard Ratio	Yes	Yes	10.1016/S0002-8703(03)00316-8
86	Ortiz-Bautista *et al*. 2020	Evaluation of a nurse-led intervention program in heart failure: A randomized trial	Medicina Clinica	10.1016/j.medcli.2018.08.005	Consisted in diet guideline, daily weighand blood pressure monitoring, HF warning clinical symptomsrecognition, flexible diuretic dosing regimen and HF treatmentadherence	Usual managementby	**Page 3:** 'Composite of all-cause mortality and all-cause hospital readmission.'	2 years	87	40	50	23	22	11	1.00	0.92	0.01	-3.81	3.82	0.6096	Hazard Ratio	Yes	Yes	https://portalfis.isciii.es/es/Paginas/inicio.aspx
87	Arashi *et al*. 2020	Long-term clinical outcomes with use of an angiotensin-converting enzyme inhibitor early after heart transplantation	American Heart Journal	10.1016/j.ahj.2020.01.003	Ramipril initiated at 2.5 mg/day	Placebo	**Page 31:** 'Composite of death from any cause, retransplantation, hospitalization for rejection or heart failure, and coronary revascularization.'	5.8 years	45	46	10	14	6	7	0.73	0.88	2.38	-12.63	17.39	0.8570	Hazard Ratio	Yes	Yes	https://clinicaltrials.gov/ct2/show/NCT01078363
88	De Luca *et al*. 2019	Final results of the randomised evaluation of short-term dual antiplatelet therapy in patients with acute coronary syndrome treated with a new-generation stent (REDUCE trial)	EuroIntervention	10.4244/EIJ-D-19-00539	Dual antiplatelet therapy (DAPT) for 3 month	Dual antiplatelet therapy (DAPT) for 12 month	**Page e991:** 'Composite occurrence of all-cause death, myocardial infarction (MI; based on the third universal definition12), stent thrombosis (ST; definite/probable, Academic Research Consortium [ARC] definition), stroke, target vessel revascularisation (TVR) and bleeding (Bleeding Academic Research Consortium [BARC] 2/3/5) within the 12-month follow-up.'	24 months	755	745	85	88	23	16	0.95	1.42	-0.14	-1.08	0.80	0.0176	Hazard Ratio	Yes	Yes	https://clinicaltrials.gov/ct2/show/NCT02118870
89	Liu *et al*. 2020	The effect of BCM guided dry weight assessment on short-term survival in Chinese hemodialysis patients	BMC Nephrology	10.1186/s12882-020-01793-x	Body composition and hydration state had been assessed by a portable whole body BIS device	Received BCM measurements at the beginning and end of the study	**Page 3:** 'Composed of death, acute myocardial infarction, cerebral infarction, cerebral hemorrhage, and peripheral vascular disease.'	13.7 months	236	209	11	19	7	11	0.51	0.56	1.16	-0.05	2.38	0.7900	Hazard Ratio	Yes	Yes	https://clinicaltrials.gov/ct2/show/NCT01509937
90	Araiza-Garaygordobil *et al*. 2020	A randomized controlled trial of lung ultrasound-guided therapy in heart failure (CLUSTER-HF study)	American Heart Journal	10.1016/j.ahj.2020.06.003	Lung ultrasound (LUS) guided therapy	Usual care	**Page 33:** 'Composite of urgent HF visits, rehospitalization for worsening HF, and death from any cause.'	6 months	63	65	20	30	9	6	0.69	1.55	-0.86	-3.40	1.69	0.1532	Hazard Ratio	Yes	Yes	https://clinicaltrials.gov/ct2/show/NCT03613779
91	de Vries *et al*. 2019	Treatment thresholds for intervention in posthaemorrhagic ventricular dilation: a randomised controlled trial	Archives of Disease in Childhood - Fetal and Neonatal Edition	10.1136/archdischild-2017-314206	Early intervention	Later intervention	**Page F2:** 'Composite of VP shunt placement and/or death.'	-	64	62	19	23	7	9	0.80	0.75	0.79	-1.41	2.98	0.8494	-	Yes	Yes	http://controlled-trials.com/ISRCTN43171322
